# Supplementary material for: Multiomic Analysis of the UV-Induced DNA Damage Response
Source: Cell Rep. 2016 May 12;15(7):1597–610. doi: 10.1016/j.celrep.2016.04.047 (PMC4893159; doi:10.1016/j.celrep.2016.04.047)
Supplement: Document S1. Supplemental Experimental Procedures and Figures S1–S5 [file mmc1.pdf]

**Cell Reports, Volume *15***

## Supplemental Information

# Multiomic Analysis of the UV-Induced DNA Damage Response

**Stefan Boeing, Laura Williamson, Vesela Encheva, Ilaria Gori, Rebecca E. Saunders, Rachael Instrell, Ozan Aygün, Marta Rodriguez-Martinez, Juston C. Weems, Gavin P. Kelly, Joan W. Conaway, Ronald C. Conaway, Aengus Stewart, Michael Howell, Ambrosius P. Snijders, and Jesper Q. Svejstrup**

**<u>Supplemental information:</u>**

**• 5 Supplementary Figures (Figure S1-S5)**

Figure S1, related to Figure 6. Screen performance and pathway enrichment.

Figure S2, related to Figure 6. Enrichment of various pathways in the data sets.

Figure S3, related to Figure 6. Enrichment of ribosome proteins is primarily caused by high scores in the ubiquitylation screens.

Figure S4, related to Figure 6 and 7. ASCC3 protein interacts with RNAPII and CSB.

Figure S5, related to Figure 7. Effect of CSB depletion on global transcription recovery after UV-irradiation.

**• Supplementary Figure Legends**

**• Supplementary Experimental Procedures**

**• 12 Supplementary Tables (Table S1-S12)**

Table S1. CSB interactomes
Table S2. C7ORF26 interactors
Table S3. RNAPII interactomes
Tables S1, S2, and S3 are related to Figure 2.

Table S4. Chromatin proteins
Table S5. Ubiquitylated proteins
Table S6. Phospho-proteome
Tables S4, S5, and S6 are related to Figure 3.

Table S7. RNAi Screen results
Table S7 is related to Figure 5.

Table S8. Ranked phospho-integration
Table S9. Scores by different approaches
Table S10. TC-NER ontology group
Table S11. Pathway enrichment
Table S12. Corum complexes enrichment
Tables S8-S12 are related to Figure 6.

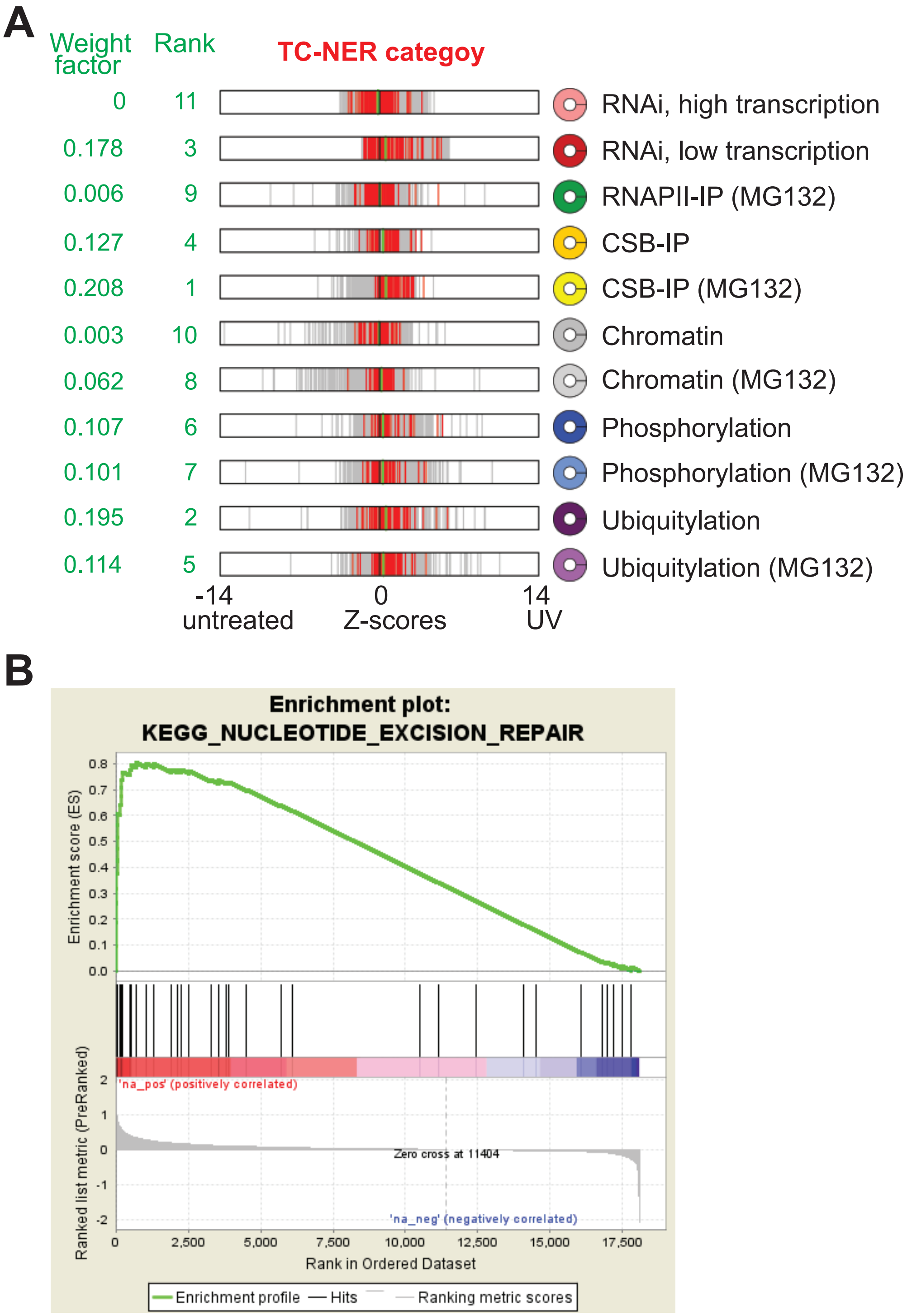
A
Weight factor
Rank
TC-NER categoy
0
11
RNAi, high transcription
0.178
3
RNAi, low transcription
0.006
9
RNAPII-IP (MG132)
0.127
4
CSB-IP
0.208
1
CSB-IP (MG132)
0.003
10
Chromatin
0.062
8
Chromatin (MG132)
0.107
6
Phosphorylation
0.101
7
Phosphorylation (MG132)
0.195
2
Ubiquitylation
0.114
5
Ubiquitylation (MG132)
-14 untreated
0 Z-scores
14 UV
B
Enrichment plot: KEGG_NUCLEOTIDE_EXCISION_REPAIR
Enrichment score (ES)
Ranked list metric (PreRanked)
'na_pos' (positively correlated)
Zero cross at 11404
'na_neg' (negatively correlated)
Rank in Ordered Dataset
Enrichment profile
Hits
Ranking metric scores

# Figure S1

High scoring pathways

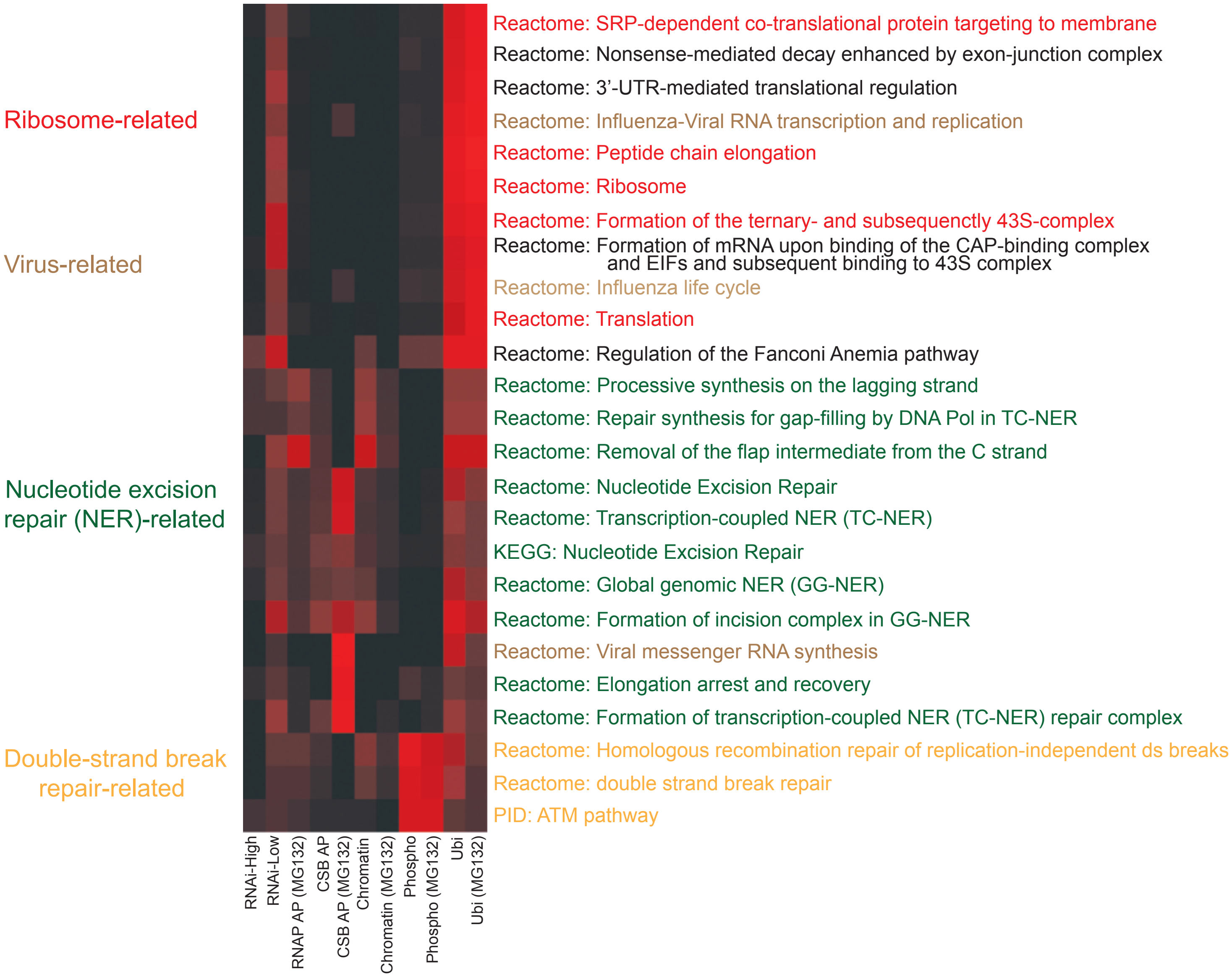


**Figure S2**

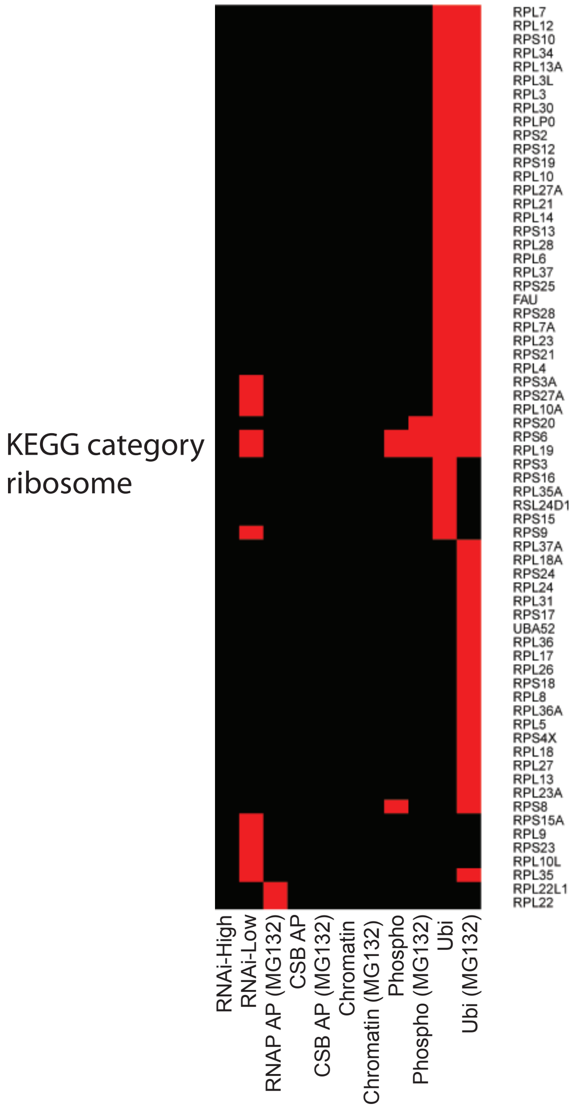
KEGG category
ribosome
RPL7
RPL12
RPS10
RPL34
RPL13A
RPL3L
RPL3
RPL30
RPLP0
RPS2
RPS12
RPS19
RPL10
RPL27A
RPL21
RPL14
RPS13
RPL28
RPL6
RPL37
RPS25
FAU
RPS28
RPL7A
RPL23
RPS21
RPL4
RPS3A
RPS27A
RPL10A
RPS20
RPS6
RPL19
RPS3
RPS16
RPL35A
RSL24D1
RPS15
RPS9
RPL37A
RPL18A
RPS24
RPL24
RPL31
RPS17
UBA52
RPL36
RPL17
RPL26
RPS18
RPL8
RPL36A
RPL5
RPS4X
RPL18
RPL27
RPL13
RPL23A
RPS8
RPS15A
RPL9
RPS23
RPL10L
RPL35
RPL22L1
RPL22
RNAi-High
RNAi-Low
RNAP AP (MG132)
CSB AP
CSB AP (MG132)
Chromatin
Chromatin (MG132)
Phospho
Phospho (MG132)
Ubi
Ubi (MG132)

**Figure S3**

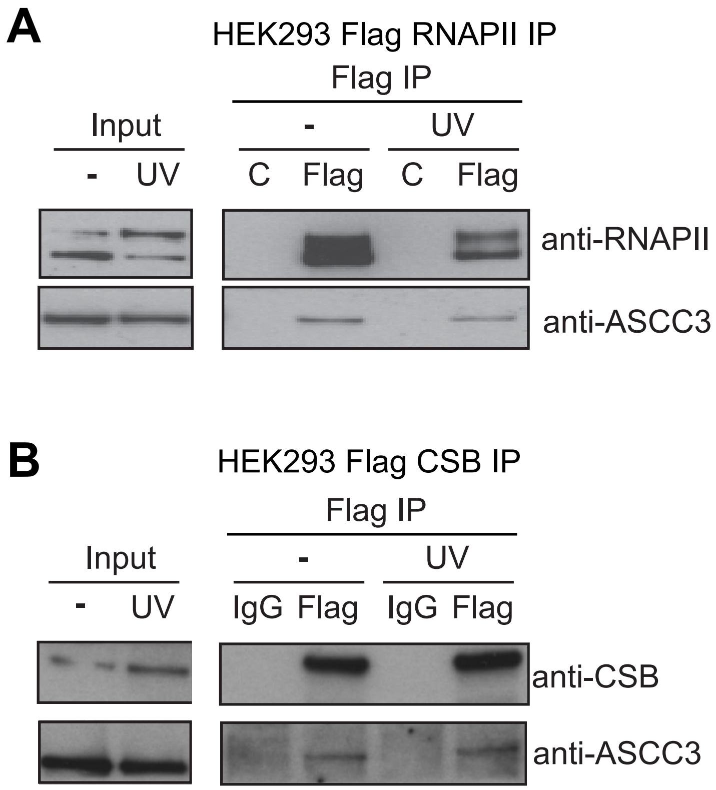
A
HEK293 Flag RNAPII IP
Flag IP
Input
-
UV
-
UV
C
Flag
C
Flag
anti-RNAPII
anti-ASCC3
B
HEK293 Flag CSB IP
Flag IP
Input
-
UV
-
UV
IgG
Flag
IgG
Flag
anti-CSB
anti-ASCC3

**Figure S4**

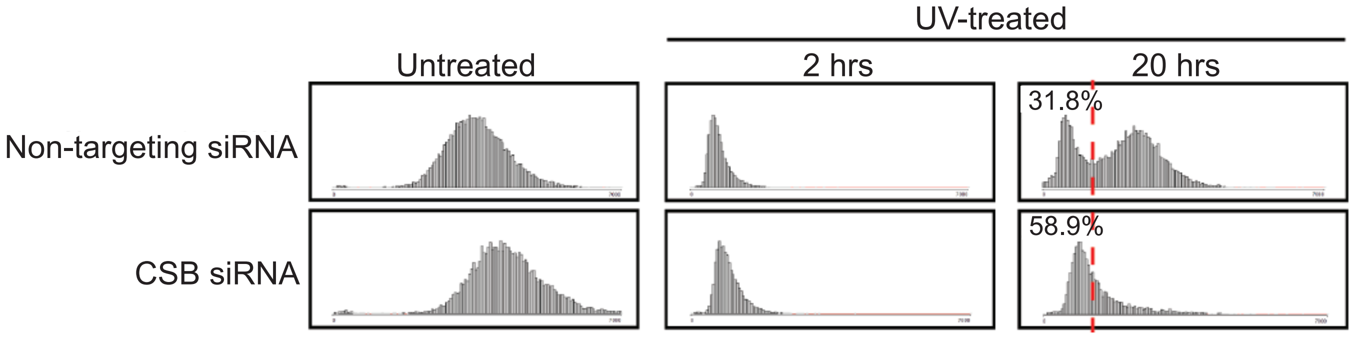
UV-treated
Untreated
2 hrs
20 hrs
Non-targeting siRNA
CSB siRNA
31.8%
58.9%

**Figure S5**

## SUPPLEMENTARY FIGURE LEGENDS

**Figure S1, related to Figure 6**. **Screen performance and pathway enrichment**. (**A**) The individual screen had different ability to uncover already known TC-NER factors. The median score and the rank of the screens are shown in green. The median score represents the frequency with which proteins from the TC-NER category scores in the indicated screen, on average. The color wheels associated with screens (on the right) are re-used in Figure 6B, C to show relative weight of that screen in the relevant score scheme. (**B**) GSEA category enrichment plot of the KEGG category 'Nucleotide_Excision_Repair' over z score sums.

**Figure S2, related to Figure 6**. **Enrichment of various pathways in the data sets.** Gene Set Enrichment Analysis (GSEA) used to illustrate the enrichment of pathways related to the ribosome, viruses, NER, and double-strand break repair. See also Table S11.

**Figure S3, related to Figure 6**. **Enrichment of ribosome proteins is primarily caused by high scores in the ubiquitylation screens.** In contrast to TC-NER and NER proteins, the significant enrichment of ribosome-related proteins is caused primarily by extremely high scores in the ubiquitylation screens, although some also score in the RNAi screen.

**Figure S4, related to Figure 6 and 7**. **ASCC3 protein interacts with RNAPII and CSB.** Immunoblot showing Flag immunoprecipitation of Flag-tagged RPB3 (**A**) and

Flag-tagged CSB (**B**) from HEK293 cells that were untreated or treated with UV-irradiation (30 J/$m^2$) followed by a 3 hour incubation. ASCC3 interacted with Flag-RPB3 and CSB equally in the absence and presence of UV-irradiation.

**Figure S5, related to Figure 7**. **Effect of CSB depletion on global transcription-recovery after UV-irradiation.** In the absence of CSB, a higher percentage of cells remain in the non-transcribing state, even 18-20 hours after DNA damage.

## Supplementary Experimental Procedures

### Cell culture, UV-irradiation

For CSB-interactomes, gly-gly peptide enrichment, and phospho-peptide enrichments, an inducible TREX 293 FLAG-CSB expressing cell line was used. FLAG-CSB expression was induced 48 hours before UV-irradiation by adding doxycycline at a concentration of 500 ng/ml to the cell culture medium. If indicated, the cell culture medium was supplemented one hour prior to the UV-treatment with the proteasome inhibitor MG132 (Calbiochem) at a concentration of 5 µM.

For RNAPII interactomes and chromatin proteomes, FLAG-RPB3 expressing HEK293 cells (Aygun et al., 2008) were used. Cells were cultured in either R0K0 (light) or R8K10 heavy SILAC medium for at least seven generations. Cells were UV-irradiated with a UV crosslinker (Stratalinker) and allowed to recover at 37°C for three hours prior to extract preparation.

For the ubiquitylome (gly-gly peptide enrichment) and phosphoproteome studies, light and heavy cells were mixed in a 1:1 ratio directly after UV-treatment.

### Extract preparation for chromatin extracts and immuno-affinity purifications

The approach taken to purify RNAPII- and CSB-interactors has been described previously (Aygun et al., 2008), with modifications outlined below. Briefly, FLAG-tagged protein was affinity-purified (AP) from Benzonase-treated chromatin, under physiological salt conditions, using anti-FLAG

antibody as previously described (Aygun et al., 2008). Specifically bound proteins were released by competition with FLAG peptide, which dramatically increased detection sensitivity and thus allowed identification of interactors that could not be detected by mass spectrometry after simple SDS-eluted FLAG immunoprecipitation (IP). As a control, we carried out IgG mock-IP experiments. Protein detection intensity was converted into iBAQ values, reflecting absolute protein abundance (Schwanhausser et al., 2011). A protein was deemed to be a specific interactor of CSB (or RNAPII below) if it was detected with at least 10-fold lower abundance in the IgG-mock AP (or not at all, as in the majority of cases). SILAC ratios were log2 transformed and then converted into z-scores in order to allow a standardized comparison to other datasets in the study.

In preparing for purification, cells were harvested in PBS and resuspended in hypotonic buffer (10 mM HEPES-KOH pH 7.5, 10 mM KCl, 1.5 mM $MgCl_2$, protease inhibitors (Roche complete), phosphatase inibitors (Roche PhosStop) and 20 mM N-Ethylmaleimide (NEM). Cells were lysed by dounce homogenization and nuclei pelleted by centrifugation at 3000x g. The cytoplasmic lysate was removed and the nuclei were resuspended in nucleoplasmic extraction buffer (20 mM HEPES pH7.9, 1.5 mM MgCl2, 10% glycerol, 150 mM potassium acetate, 0.05% NP-40, protease and phosphatase inhibitors). After a 20 min incubation on ice, chromatin was pelleted by centrifugation at 20,000x g. The nucleoplasmic lysate was removed and the pellet resuspended in chromatin digestion buffer (20 mM HEPES-KOH 7.9, 1.5 mM $MgCl_2$, 10% glycerol, protease and phosphatase inhibitors as above). The pan-nuclease Benzonase (EMD Millipore) was

added to a concentration of 75 units/ml and nucleic acids digested at 37°C for 15 min. The salt concentration was then increased to 150 mM NaCl by adding a 5 M NaCl solution, dropwise. The NP-40 concentration was similarly brought to 0.05%, and the solution was incubated on ice for 20 min. Debris was then pelleted by centrifugation at 20,000x g and the supernatant collected as chromatin fraction.

All immunoprecipitations in this study were performed using the chromatin fraction. Mouse-IgG coupled beads were used alongside FLAG-M2 beads (Sigma-Aldrich) in each experiment for specificity-control. Both kinds of beads were incubated for 3 hrs at 4°C with chromatin extracts from either FLAG-CSB or FLAG-RPB3 expressing cell lines. AP beads were then washed three times with 50 column-volumes AP-wash buffer (20 mM HEPES pH 7.9, 1.5 mM $MgCl_2$, 10% glycerol, 150 mM NaCl, 0.05% NP-40, protease and phosphatase inhibitors as above), and subsequently eluted with 3xFLAG peptide at 1 mg/ml.

**Western blotting**

Antibodies for SCAF4 and SCAF8 were purchased from Bethyl, the INTS4 antibody from abcam and the UVSSA antibody from Novus Biologicals. RNAPII CTD serine-2, -5, -7, CTD threonine-4, and CTD tyrosine-1 antibodies were kind gifts of Professor Dirk Eick, University of Munich.

**Quantitative diGly Proteomics**

Cells were lysed in 9 M urea, 20mM HEPES pH 8.0, supplemented with 100 units/ml of benzonase and sonicated to reduce viscosity (3mm probe, 50 %

amplitude, 3 x 15 sec bursts, on ice). Between 30 - 40 mg of protein per sample were used as estimated by Bradford protein assay. Lysates were reduced with 10 mM dithiothreitol (DTT) (Sigma) for 30 min at room temperature, followed by alkylation with 20 mM chloroacetamide (Sigma) for 30 min at room temperature in the dark. Lysates were digested initially with LysC (Promega) for 2 hours at $37^0$C. The lysates were then diluted with 100 mM amonium bicarbonate, 5 % acetonitrile to a final urea concentration of less than 2 M. The samples were digested 1:100 enzyme to protein ratio (w/w) with trypsin (Promega) overnight at $37^0$C. The next day, two additional aliquots of trypsin were added and incubated at 37 $^0$C four hours each. After the digestion the samples were acidified with TFA (Thermo Fisher Scientific) to final concentration of 1 % (v/v). All insoluble material was removed by centrifugation and the supernatant was desalted with Sep-Pak $C_{18}$ cartridges (Waters) and lyophilized for 2 days.

Peptides containing the diGly remnant were enriched using K-ϵ-GG affinity resin (Cell Signaling Technology) according to the manufacturer's instructions. Briefly, digests were reconstituted in 1.4 ml of immunoaffinity purification (IAP) buffer as supplied by the manufacturer. One aliquot (~40-μl packed bead volume) was washed four times with PBS and mixed with the peptide sample. Incubation of sample and beads was performed with gentle rotation at 4 °C for 2 hours followed by a 30 sec 2000 × *g* spin to pellet the beads. The antibody beads were washed twice with ice-cold IAP buffer followed by three washes with ice-cold water. DiGly peptides were eluted from the beads with the addition of 50 μl of 0.15 % TFA and allowed to stand at room temperature for 5 min. After a 30 sec 2000 × *g* spin, the supernatant was carefully removed

and retained for further analysis. A second 55-µl aliquot of 0.15 % TFA was added to the beads followed by a 30 sec 2000 × *g* spin, and the supernatant was added to the first elution. The eluted peptides were lyophilised for 2 days and used for SCX fractionation.

**SCX (strong cationic exchange) fractionation of diGly peptides**

For SCX fractionation, peptides eluted from the K-ϵ-GG affinity resin were dissolved in 35 µl of 10 mM Ammonium formate pH 2.9, 25 % acetonitrile. The samples were sonicated and insoluble material was removed by centrifugation. Peptide separation and fraction collection was performed using the micro pump on a RSLCnano U3000 (Thermo Fisher Scientific) at a flow rate of 50 µl/min. The peptides were loaded on 15-cm Polysulfoethyl-Asp SCX column (1 mm inner diameter, 5 µm particle size, PolyLC). Solvent A was 10 mM Ammonium formate pH 2.9, 25 % acetonitrile, and solvent B was 500 mM Ammonium formate pH 6.8, 25 % acetonitrile. The samples were run on a linear gradient of 0-80 % B in 45 min, total run time was 75 min including column conditioning. A total of 30 fractions were collected every minute between 15-45 min after injection (1 fraction= 50 µl). The collected fractions were vacuum dried and used for LC-MS/MS analysis.

**SCX fractionation of phosphopeptides**

Peptides were resuspended in 90 µl of 10 mM Ammonium formate pH 2.9, 25 % acetonitrile, sonicated and all insoluble material was removed by centrifugation. Separation was performed using Agilent 1200 (Agilent) HPLC at a flow rate of 1 ml/min. The peptides were loaded on 20-cm Polysulfoethyl-

Asp SCX column (4.6 mm inner diameter, 5 µm particle size, PolyLC). Solvent A was 10 mM ammonium formate pH 2.9, 25 % acetonitrile, and the solvent B was 500 mM ammonium formate pH 6.8, 25 % acetonitrile. The samples were run on a linear gradient 0-80 % B in 10 min. The total run time including column conditioning was 30 min. A total of six fractions were collected every two min between 1-13 min after injection (1 fraction = 2 ml). The collected fractions were vacuum dried and used for phosphopeptide enrichment.

**TiO2 enrichment of phosphopeptides**

Dried fractions were solubilised in 1 ml of loading buffer (80 % acetonitrile, 5 % TFA, 1M glycolic acid), sonicated and mixed with 5 mg of $TiO_2$ beads (Titansphere, 5 µm GL Sciences Japan). Samples were incubated for 10 min with agitation followed by a 1 min 2000 × *g* spin to pellet the beads. The supernatant was removed and the beads were washed with 150 µl of loading buffer. This was followed by two wash steps, first with 150 µl 80 % acetonitrile, 1 % TFA and second with identical volume of 10 % acetonitrile, 0.2 % TFA. After each wash beads were pelleted by centrifugation (1 min at 2000 × *g*) and the supernatant discarded. The remaining solution and beads were dried in a vacuum centrifuge for 30 min followed by two elution steps at high pH. For the first elution step the beads were mixed with 100 µl of 1 % ammonium hydroxide (v/v), incubated for 10 min with agitation and pelleted at 2000 × *g* for 1 min. For the second elution step the beads were mixed with 100 µl of 5 % ammonium hydroxide (v/v), incubated for 10 min with agitation and pelleted at 2000 × *g* for 1 min. The two elutions were combined, vacuum dried and desalted using C18 Stage Tips (Rappsilber *et al*, 2007, Nat. Prot).

Briefly, each Stage Tip was packed with one C18 Empore™ high performance extraction disk and conditioned with 100 µl of 100 % methanol, followed by 200 µl of 1 % TFA. The sample was loaded on the conditioned Stage Tip, washed six times with 200 µl of 1 % TFA and elute with 50 µl of 80 % acetonitrile, 5 % TFA. The desalted peptides were vacuum dried in preparation for LC-MS/MS analysis.

**In-gel digestion**

Polyacrylamide gel slices were prepared for mass spectrometric analysis using the Janus liquid handling system (Perkin-Elmer). Briefly, the excised protein gel piece was placed in a well of a 96-well microtiter plate and destained with 50 % acetonitrile, 50 mM ammonium bicarbonate, reduced with 10 mM DTT, and alkylated with 55 mM iodoacetamide. After alkylation, the proteins were digested with 6 ng/µl trypsin overnight at 37°C. The resulting peptides were extracted in 2 % formic acid, 1 % acetonitrile.

**LC-MS/MS**

For MS analysis, peptides were resuspended in 0.1 % TFA and loaded on 50-cm Easy Spray PepMap column (75 µm inner diameter, 2 µm particle size, Thermo Fisher Scientific) equipped with an integrated electrospray emitter. Reverse phase chromatography was performed using the RSLC nano U3000 (Thermo Fisher Scientific) with a binary buffer system at a flow rate of 250 nl/min. Solvent A was 0.1 % formic acid, 5 % DMSO, and solvent B was 80 % acetonitrile, 0.1 % formic acid, 5 % DMSO. The diGly enriched samples and the in-gel digested samples (RNA polymerase IP, CSB IP and chromatin

proteome) were run on a linear gradient of solvent B (2- 40 %) in 90 min, total run time of 120 min including column conditioning. The phopsho-enriched samples were run on a linear gradient of 2-35 % B in 150 min, total run time of 186 min. The nanoLC was coupled to a Q Exactive mass spectrometer using an EasySpray nano source (Thermo Fisher Scientific).

The Q Exactive was operated in data-dependent mode acquiring HCD MS/MS scans (R=17,500) after an MS1 scan (R=70, 000) on the 10 most abundant ions using MS1 target of $1 \times 10^6$ ions, and MS2 target of $5 \times 10^4$ ions. The maximum ion injection time utilized for MS2 scans was 120 ms, the HCD normalized collision energy was set at 28, the dynamic exclusion was set at 10 s, and the peptide match and isotope exclusion functions were enabled.

The phospho-enriched samples were also analysed using a LTQ Orbitrap Velos (Thermo Fisher Scientific) where the HPLC conditions were as described above. CID and Multi Stage Activation (MSA) fragmentation were used in separate runs to increase the total number of identified phosphopeptides. The LTQ Orbitrap Velos was operated in data-dependent mode acquiring 10 CID or MSA MS2 scans (R=17,500) after an MS1 scan (R=60, 000). MS1 target was set at $1 \times 10^6$ ions, and MS2 target at $3 \times 10^4$ ions. The CID normalized collision energy was set at 35 with 10 ms activation time and a maximum ion injection time for MS2 scans at 50 ms. The dynamic exclusion was set at 20 s and singly charged peptides and peptides with unassigned charge states were excluded from fragmentation.

## Proteomics data processing and analysis

Raw data files were analysed with MaxQuant software (version 1.3.0.5) as described previously (Cox et al., 2009). Parent ion and tandem mass spectra were searched against UniprotKB *Homo sapiens* database (August 2012). A list of 247 common laboratory contaminants provided by MaxQuant was also added to the database. For the search the enzyme specificity was set to trypsin with maximum of three missed cleavages for the diGly dataset and two missed cleavages for the rest of the data. The precursor mass tolerance was set to 20 ppm for the first search (used for mass re-calibration) and to 6 ppm for the main search. Carbamidomethylation of cysteines was specified as fixed modification, oxidized methionines and N-terminal protein acetylation were searched as variable modifications. Di-glycine-lysine or phosphorylated serine, threonine and tyrosine were added to the list of variable modifications when samples enriched for ubiquitinated or phosphorylated peptides were searched. The datasets were filtered on posterior error probability to achieve 1 % false discovery rate on protein, peptide and site level.

Proteomic raw data were processed using the MAXQUANT software package (Cox et al., 2009). Log2-transformed Heavy to Light SILAC ratios (Maxquant output) were converted into z-scores as follows:

SILAC Z-score = [log2(SILAC Ratio H/L) – mean of all log2(SILAC Ratio H/L)] / standard deviation (all log2(SILAC Ratio H/L)). Ensembl gene identifiers were added using the PERSEUS software package (Cox and Mann, 2012). Based on the Ensembl gene identifiers, official human genome nomenclature (HGNC) gene names were added. Phosphorylation and Ubiquitylation data was processed in a similar way. Official HGNC gene symbols were assigned to siRNAs data using Ensembl gene identifiers.

Individual proteomic and RNAi datasets were intersected using ensemble gene identifiers. Aggregated z-scores were calculated by adding up the z-scores of the individual experiments. In case a protein had more than one phosphorylation or more than one ubiquitylation site in any one experiment, only the highest scoring phosphorylation and/or ubiquitylation site was considered for that particular experiment when calculating aggregated z-scores.

**RNAi Screen**

Dharmacon Human siGENOME library was aliquoted into 384-well plate format. Each well contained a pool of 4 siRNAs targeting a single gene. siRNAs targeting CSB and the RPB1 subunit of RNAPII were included as positive controls and RISC-free and non-targeting pool 2 as negative controls. siRNA pools diluted in HBSS (Invitrogen) (5μL/well, final concentration 37.5 nM) and the transfection reagent INTERFERin (Polyplus-transfection) diluted in Opti-MEM (Invitrogen) (5 μL/well, final concentration of 0.05 μl/well) were deposited in clear-bottomed 384-well plates (Greiner Bio One). After 15 min incubation at room temperature, MRC5VA cells were diluted in DMEM supplemented with 10% fetal bovine serum and 5% penicillin/streptomycin and deposited on top of the transfection mix (900 cells/well) to give a final volume of 50 μL per well. Plates were incubated at 37°C and 5% $CO_2$ for 48 hour. Media was removed from the plates and the plates were exposed to UVC light (TUV 16W G16 T5, Philips) using a purpose built UV irradiation machine that enabled an even exposure of UV light across the plate (stationary bulb coupled to a conveyor belt that moved the plate

under the bulb in a constant manner). Media was then added back onto the plates and they were incubated for a subsequent 18 hours. Media was then removed from the plates and replaced with fresh media containing 0.75 mM 5'-ethynyl uridine (Jena Bioscience). Plates were incubated with 5'-ethynyl uridine containing media for 2 hour to allow for uptake into the cells and incorporation in the nascent RNA. Media was then removed and the cells were fixed with 3.7% formaldehyde (Sigma) for 60 min at room temperature followed by one wash with PBS and permeabilization using 0.5% TX-100 for 30 min. Plates were then washed once with PBS followed by incubation with staining solution (100 mM Tris pH 8.5 (Sigma), 4 mM $CuSO_4$ (Sigma), 10 µM AlexaFlour azide 488 nM (Invitrogen), 100 mM ascorbic acid (Sigma)) for 60 min at room temperature. Plates were washed 3 times in Tris pH7.5 and stained with DAPI (4′,6-diamidino-2-phenylindole dihydrochloride, Invitrogen) at a final concentration of 1 µg/ml. Plates were washed once with PBS. The screen was performed in triplicate. Automated image acquisition of 6 fields per well was performed on a Cellomics Array Scan VTI HCS reader (Thermo Scientific) using a 10× objective. Image analysis was performed using HCS Studio (Thermo Scientific). Cell nuclei were masked using the DAPI staining. The average intensity of AlexaFluor-conjugated 5' ethynyl uridine-labeled RNA was measured for each nucleus. The high transcription score was calculated by taking the average fluorescence intensity per nuclei per well for each replicate and then normalizing to the plate median. The normalised values were then converted to a z-score and the median of the 3 replicate z scores was used as the final score. We set an arbitrary cutoff of z= 2.0 to classify siRNA hits as high transcribers. The low transcription score was

calculated by determining the percentage of cells per well below a defined threshold and normalizing it to the plate median. The normalized values were then converted to a z score and the median of the 3 replicate z scores was used as the final score. We set an arbitrary cutoff of z=2.5 to classify siRNA hits as low transcribers.

**Statistical evaluation**

Category enrichment p-values for pathways and protein complexes were calculated using the Kolmogorov-Smirnov test.

**Data sources**

Protein complex data was retrieved from CORUM (Ruepp et al., 2010), protein-protein interaction data from biogrid (Stark et al., 2006), protein domain data from INTERPRO (http://www.ebi.ac.uk/interpro), pathways were taken from Reactome (http://www.reactome.org), the pathway interaction database (http://pid.nci.nih.gov) KEGG (http://www.genome.jp/kegg/pathway.html) and mySigDB (Subramanian et al., 2005).

**Aggregate scores**

Point scores: Each gene earned one point for each screen in which it had scored above the z-score cut-off. For the RNAi low screen that z- score cut off was set arbitrarily to 2.5; for the RNAi high screen to 2 and for all proteomic screens to 1.5. If a gene featured more than one phosphorylation and/or ubiquitylation site, only the highest scoring site was used to calculate the z-

score sum for that gene in the respective screen. The median score among proteins of the TC-NER reference category in the point scoring scheme was 1 using this approach (compared to 0 for a list of all curated proteins), indicating an enrichment of the benchmark category in the overall data (Category enrichment p-value=$1.69*10^{-14}$).

Z-score sums: Z-score sums were calculated by adding up the z-scores of individual screens for each gene. In case a gene had not featured in a particular screen, 0 was added to the total z-score sum. If a gene featured more than one phosphorylation and/or ubiquitylation site, only the highest scoring site was used to calculate the z-score sum for that gene in the respective screen. The median of the TC-NER benchmark category in this metric was at 0.17, indicating an enrichment of this category (category enrichment p-value < $2.2*10^{-16}$).

Weighted z-score sums: For the weighted z-score sums the z-scores of the individual screens were multiplied with a weight factor proportional to the scores of the TC-NER training category in that screen. The weight factors were as follows: RNAi high screen: 0; RNAi low screen: 0.178; RNAPII-IP(MG132): 0.006; CSB-IP: 0.127; CSB-IP(MG132): 0.208; chromatin MS: 0.003; chromatin MS(MG132): 0.062; phosphorylation: 0.107; phosphorylation (MG132): 0.101; ubiquitylation: 0.195; ubiquitylation(MG132): 0.114. Weighted z-scores were summed up for each gene in the same way as for the z-score sums above. In this metric the median of the TC-NER benchmark

category was 0.41, the median score for all other genes was 0.02 (category enrichment p-value < $2.2*10^{-16}$)

**Protein Kinase networks**

In order to infer possible kinase-substrate relationships, we took advantage of curated protein-protein interaction data in the Biogrid database (www.thebiogrid.org). Based on this, we created a protein-protein interaction network and analyzed if proteins, that are directly connected to a protein kinase exhibit an increased phosphorylation status (defined as a z-score of 1.5 or bigger in at least one of the phosphor proteomic experiments) upon UV-irradiation.

**Laser micro-irradiation microscopy**

Cells were plated in gridded MatTek dishes (35mm, No. 2 14mm diameter glass) at a density of 2 x $10^6$ per dish in media containing DMEM + 10% FCS + 100 µg/ml Hygromycin B + 15 µg/ml blasticidin supplemented with 5 µg/mL doxycycline hyclate (Sigma-Aldrich D9891) for 48 hours. 30 min prior to imaging, culture medium was replaced with phenol-red free medium containing the same additives plus 1 µg/ml Hoechst 33258 to label nuclei and sensitize cells to UV irradiation.

UV-microirradiation was performed by subjecting cell nuclei to laser micro-irradiation in a 200 x 3 pixel (34 x 0.51 µm) stripe or a diffraction limited spot. Micro-irradiation was performed with 100% 405 nm laser power and cells were exposed to 500-700 µW for approximately 3s (40 iterations) and

1.5s (250 iterations) for the stripe and spot, respectively. Normal cell and nuclear morphology were preserved over the time scale of the experiment.

Micro-irradiation and imaging was performed on a Perkin Elmer UltraVIEW VoX spinning disk microscope, which included a Yokagawa CSU-X11 spinning disk, an ORCA-R2 camera (Hamamatsu), and a Perkin Elmer PhotoKinesis accessory. The microscope base was a Carl Zeiss Axiovert 200M equipped with a 40x 1.3 NA Plan-Apochromat objective and a 37$^{o}$ C, 5% $CO_2$ incubator (Solent Scientific). STK19-GFP was excited with the 488 nm laser and imaged with a 500-550 emission filter. Laser power and exposure time were adjusted before-hand to maximize image quality and minimize photobleaching; absence of significant photobleaching was confirmed by observing unperturbed cells in the acquisition field of view.

**References:**


Aygun, O., Svejstrup, J., and Liu, Y. (2008). A RECQ5-RNA polymerase II association identified by targeted proteomic analysis of human chromatin. Proceedings of the National Academy of Sciences of the United States of America *105*, 8580-8584.

Cox, J., and Mann, M. (2012). 1D and 2D annotation enrichment: a statistical method integrating quantitative proteomics with complementary high-throughput data. BMC Bioinformatics *13 Suppl 16*, S12.

Cox, J., Matic, I., Hilger, M., Nagaraj, N., Selbach, M., Olsen, J.V., and Mann, M. (2009). A practical guide to the MaxQuant computational platform for SILAC-based quantitative proteomics. Nat Protoc *4*, 698-705.

Ruepp, A., Waegele, B., Lechner, M., Brauner, B., Dunger-Kaltenbach, I., Fobo, G., Frishman, G., Montrone, C., and Mewes, H.W. (2010). CORUM: the comprehensive resource of mammalian protein complexes--2009. Nucleic acids research *38*, D497-501.

Schwanhausser, B., Busse, D., Li, N., Dittmar, G., Schuchhardt, J., Wolf, J., Chen, W., and Selbach, M. (2011). Global quantification of mammalian gene expression control. Nature *473*, 337-342.

Stark, C., Breitkreutz, B.J., Reguly, T., Boucher, L., Breitkreutz, A., and Tyers, M. (2006). BioGRID: a general repository for interaction datasets. Nucleic acids research *34*, D535-539.

Subramanian, A., Tamayo, P., Mootha, V.K., Mukherjee, S., Ebert, B.L., Gillette, M.A., Paulovich, A., Pomeroy, S.L., Golub, T.R., Lander, E.S., *et al.* (2005). Gene set enrichment analysis: a knowledge-based approach for interpreting genome-wide expression profiles. Proceedings of the National Academy of Sciences of the United States of America *102*, 15545-15550.
