# Supplementary material for: Multiomic Analysis of the UV-Induced DNA Damage Response
Source: Cell Rep. 2016 May 12;15(7):1597–610. doi: 10.1016/j.celrep.2016.04.047 (PMC4893159; doi:10.1016/j.celrep.2016.04.047)
Supplement: Document S2. Article plus Supplemental Information [file mmc14.pdf]

Resource

Cell Reports

Multiomic Analysis of the UV-Induced DNA Damage Response

Graphical Abstract

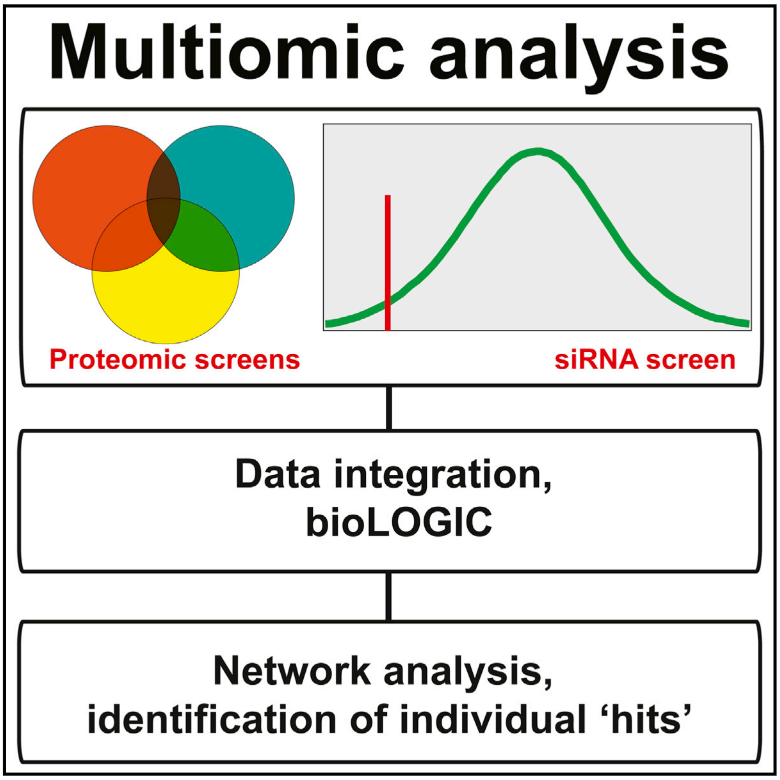

Highlights

- A multiomic screening approach examines the UV-induced DNA damage response
- Multiple factors are connected to the transcription-related DNA damage response
- Melanoma gene STK19 is required for a normal DNA damage response

Authors

Stefan Boeing, Laura Williamson, Vesela Encheva, ..., Michael Howell, Ambrosius P. Snijders, Jesper Q. Svejstrup

Correspondence

jesper.svejstrup@crick.ac.uk

In Brief

Boeing et al. investigate the UV-induced DNA damage response by combining a range of proteomic and genomic screens. A function in this response for the melanoma driver STK19 as well as a number of other factors are uncovered.

Boeing et al., 2016, Cell Reports *15*, 1597–1610
May 17, 2016 © 2016 The Author(s)
http://dx.doi.org/10.1016/j.celrep.2016.04.047

Cell Reports
Resource

OPEN ACCESS
CellPress

Multiomic Analysis of the UV-Induced DNA Damage Response

Stefan Boeing,[1,5] Laura Williamson,[1] Vesela Encheva,[2] Ilaria Gori,[3] Rebecca E. Saunders,[3] Rachael Instrell,[3] Ozan Aygün,[1,7] Marta Rodriguez-Martinez,[1] Juston C. Weems,[4] Gavin P. Kelly,[5] Joan W. Conaway,[4,6] Ronald C. Conaway,[4,6] Aengus Stewart,[5] Michael Howell,[3] Ambrosius P. Snijders,[2] and Jesper Q. Svejstrup[1,*]
[1]Mechanisms of Transcription Laboratory, the Francis Crick Institute, Clare Hall Laboratories, South Mimms EN6 3LD, UK
[2]Protein Analysis and Proteomics Laboratory, the Francis Crick Institute, Clare Hall Laboratories, South Mimms EN6 3LD, UK
[3]High Throughput Screening Laboratory, the Francis Crick Institute, 44 Lincoln's Inn Fields, London WC2A 3LY, UK
[4]Stowers Institute for Medical Research, Kansas City, MO 64110, USA
[5]Bioinformatics and Biostatistics Laboratory, the Francis Crick Institute, 44 Lincoln's Inn Fields, London WC2A 3LY, UK
[6]Department of Biochemistry and Molecular Biology, University of Kansas Medical Center, Kansas City, KS 66160, USA
[7]Present address: Department of Chemistry, Massachusetts Institute of Technology, Cambridge, MA 02139, USA
*Correspondence: jesper.svejstrup@crick.ac.uk
http://dx.doi.org/10.1016/j.celrep.2016.04.047

SUMMARY

In order to facilitate the identification of factors and pathways in the cellular response to UV-induced DNA damage, several descriptive proteomic screens and a functional genomics screen were performed in parallel. Numerous factors could be identified with high confidence when the screen results were superimposed and interpreted together, incorporating biological knowledge. A searchable database, bioLOGIC, which provides access to relevant information about a protein or process of interest, was established to host the results and facilitate data mining. Besides uncovering roles in the DNA damage response for numerous proteins and complexes, including Integrator, Cohesin, PHF3, ASC-1, SCAF4, SCAF8, and SCAF11, we uncovered a role for the poorly studied, melanoma-associated serine/threonine kinase 19 (STK19). Besides effectively uncovering relevant factors, the multiomic approach also provides a systems-wide overview of the diverse cellular processes connected to the transcription-related DNA damage response.

INTRODUCTION

The cellular response to bulky DNA lesions, such as those induced by UV irradiation is multi-faceted. The effect of such damage on transcription is particularly complex. Bulky DNA lesions in the transcribed strand cause stalling of RNA polymerase II (RNAPII), resulting in a block to transcript elongation. Damage-stalled RNAPII then functions as a molecular beacon that triggers transcription-coupled nucleotide excision repair (TC-NER), the process whereby DNA damage in the transcribed strand of active genes is preferentially removed (Gaillard and Aguilera, 2013). On the other hand, if the DNA lesion for some reason cannot be removed by TC-NER, a mechanism of last resort ensures that RNAPII is ubiquitylated and degraded by the proteasome, enabling repair by other mechanisms (Wilson et al., 2013).

Importantly, bulky DNA lesions not only block RNAPII progress, but also affect transcription genome-wide so that even un-damaged genes temporarily cease to be expressed (Mayne and Lehmann, 1982; Rockx et al., 2000; Proietti-De-Santis et al., 2006). The mechanisms and factors that underlie TC-NER and the more general DNA-damage-induced repression of gene expression are still poorly understood.

Cockayne syndrome B protein (CSB, also named ERCC6) plays a key role in both TC-NER and the global transcription response to DNA damage (Vermeulen and Fousteri, 2013). CSB is recruited to damage-stalled RNAPII, allowing assembly of the core NER machinery around it (Fousteri et al., 2006). CSB is also required for the subsequent DNA incisions, permitting lesion removal (Anindya et al., 2010). Importantly, CSB additionally helps regulate global RNAPII-mediated transcription. Indeed, CSB is crucial for the general recovery of transcription after DNA damage (Mayne and Lehmann, 1982), in a process that is partly independent of its role in repair (Rockx et al., 2000; Proietti-De-Santis et al., 2006). CSB contains a functionally important ubiquitin-binding domain (Anindya et al., 2010) and is itself both ubiquitylated (Groisman et al., 2003; Groisman et al., 2006) and phosphorylated (Christiansen et al., 2003), supporting the idea that post-translational modifications (PTMs) are important in the DNA damage response. Some CSB ubiquitylation is carried out by a ubiquitin ligase complex containing CSA (Groisman et al., 2006), a TC-NER factor that transfers to chromatin only after DNA damage (Kamiuchi et al., 2002).

With these factors and mechanisms in mind, we set out to chart the transcription-related DNA damage response. In modern "global screens," the characteristics of thousands of proteins or genes can be mapped concomitantly, but it is often problematic to recognize the important candidates in a list of hundreds of scoring proteins. In the hope of addressing this difficulty, we developed a multiomic approach. In this approach, distinct global screens were performed under the same conditions and the results then overlapped and integrated. Specifically, we used quantitative proteomics to determine the impact

This is an open access article under the CC BY license (http://creativecommons.org/licenses/by/4.0/).

OPEN
ACCESS
CellPress

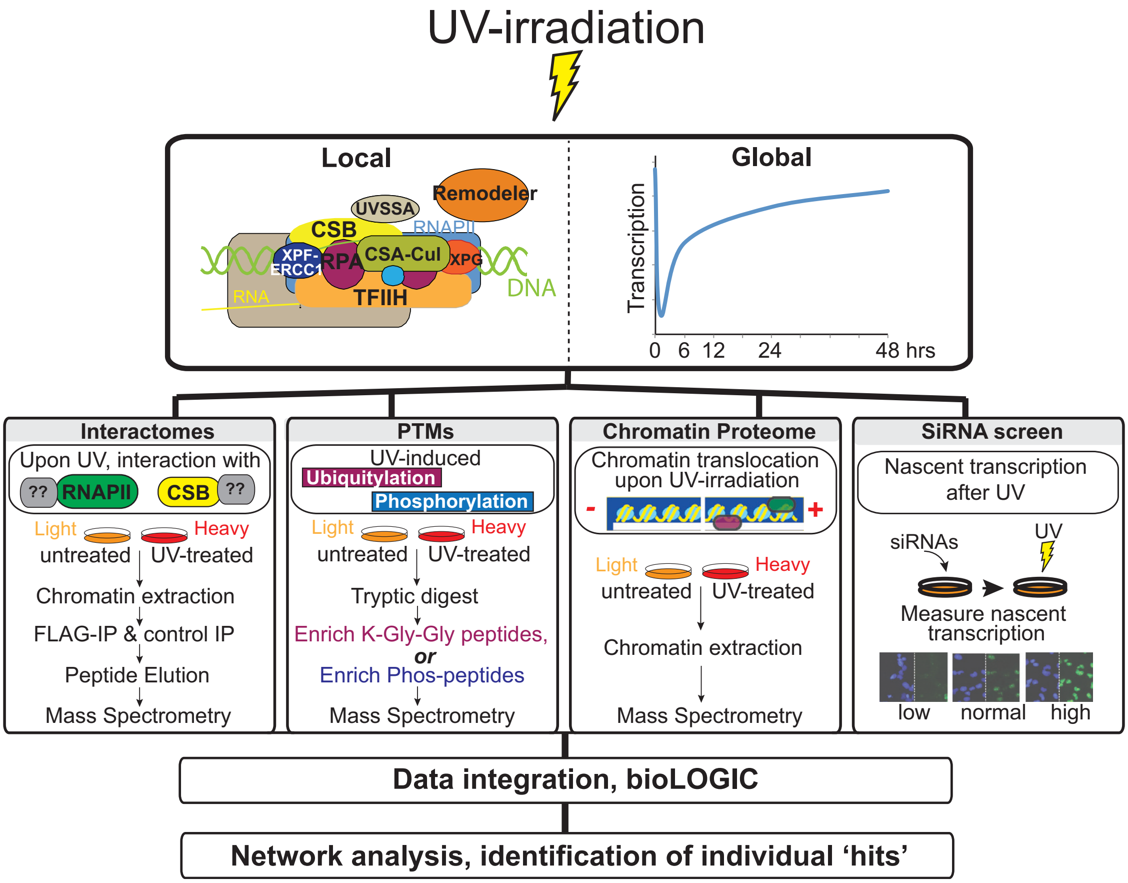

Figure 1. Graphical Overview of the Multiomic Approach to Charting the Transcription-Related DNA Damage Response

UV-induced DNA damage has effects both at the local ("repairosome") and the global level. The proteomic screens and the siRNA screen used to investigate the damage response are outlined. UV irradiation (30 J/m^2) was used for all proteomic analysis, while 15 J/m^2 was used in the RNAi screen.

of DNA damage on (1) the RNAPII interactome, (2) the CSB interactome, (3) chromatin association dynamics, (4) the protein ubiquitylome, and (5) the phosphoproteome. This was complemented with (6) a functional RNAi screen. Candidates were then ranked based on their performance in the screens overall and further filtered for biological relevance and technical robustness and are searchable using a newly established database, named bioLOGIC. The multiomic approach not only confirmed the involvement of well-known TC-NER factors, but also uncovered numerous new factors and cellular pathways that have not previously been connected to the transcription-related DNA damage response. One example is the poorly studied melanoma gene STK19.

RESULTS

To uncover factors with a role in the transcription-related DNA damage response, we carried out a combination of proteomic and genomic screens. The UV-induced DNA damage response has typically been studied at early time-points (30 min to 1 hr after UV exposure), but in order to also gain insight at the recovery phase, we performed all proteomics screens with material extracted from HEK293 cells at 3 hr after UV-induced DNA damage. We hoped this would uncover factors across the whole DNA damage response, from early events, such as DNA damage signaling and gene expression shutdown, to late events, such as post-incision repair factors and transcription-recovery proteins (see Figure 1). The proteomic screens were performed under identical conditions and all made use of quantitative stable isotope labeling by amino acids in cell culture (SILAC) proteomics (Ong et al., 2002), enabling us to distinguish between "constitutive" and UV-induced interactors and modifications. Moreover, proteasome inhibition has previously been shown to prevent dissociation of certain DNA-damage-induced protein interactions (Groisman et al., 2006). We therefore also carried out all proteomic experiments in the presence of proteasome inhibitor MG132.

CSB Interactome

CSB is the central transcription-repair coupling factor and is specifically recruited to damage-stalled RNAPII. The UV-induced CSB interactome was evaluated, starting from chromatin (Aygün et al., 2008). Numerous proteins became recruited to CSB in response to UV irradiation, with the identification of TC-NER

factors such as UVSSA, the CSA-ubiquitin ligase complex (CUL4/DDB1/CSA), and the core transcription factor II H (TFIIH) complex validating the screen.

Excitingly, several other interesting interactions were detected (Figure 2A; Table S1) (see also the searchable database at http://www.biologic-db.org [username: guest, password: guest01]). Only a few interactions are highlighted here. For example, the WDR82/PPP1R10/TOX4 complex was recruited to CSB upon DNA damage. This complex recognizes DNA adducts generated by platinum anticancer drugs (Bounaix Morand du Puch et al., 2011), but a role in the UV damage response has not previously been reported. The Integrator complex, previously linked to small nuclear RNA maturation and more generally to RNAPII transcription (Baillat and Wagner, 2015), was strongly recruited as well. Interestingly, ASUN, C7ORF26, VWA9/C15orf44, DDX26B, and NABP1/2 were recruited with strikingly similar proteomic characteristics to those of the "canonical" integrator complex subunits (Baillat et al., 2005), supporting the idea that they are de facto Integrator subunits (Malovannaya et al., 2010). Indeed, immunoprecipitation (IP) of FLAG-tagged C7ORF26 brought down all these proteins, except for NABP1 (Table S2). NABP1 is part of the so-called sensor of ssDNA (SOSS) complex, which participates in ATM kinase activation and repair of DSBs and contains the Integrator subunits INTS6, DDX26B, and INTS3 (Zhang et al., 2013 and references therein). Our data thus raise the interesting possibility that a complete, NABP1-containing Integrator "super-complex" is recruited to CSB upon UV irradiation.

Surprisingly, TERF2 (otherwise known as TRF2) and the TERF2-interacting protein TERF2IP (otherwise known as RAP1) were also recruited upon UV irradiation. Although predominantly studied as telomeric proteins, TERF2 and TERF2IP have previously been implicated in a general response to DNA double-strand breaks (Bradshaw et al., 2005; Williams et al., 2007), but a connection to the UV damage response has not been reported. Several other factors, such as PHF3, SETD2, PCF11, CDK9, SCAF4/SCAF8, the CTD phosphatase regulator and human homolog of yeast Rtt103, RPRD1B (Morales et al., 2014; Ni et al., 2014), as well as TCEB3/Elongin A1, were markedly recruited to CSB after UV irradiation as well (Table S1).

In the cases where it was tested, IP-western experiments confirmed these results (Figure 2B).

RNAPII Interactome

We similarly examined the changes in the RNAPII interactome upon UV irradiation (Figure 2C; Table S3). RNAPII is ubiquitylated and degraded upon DNA damage, so for this screen we only cultured cells in the presence of proteasome inhibitor MG132.

All RNAPII interactors previously detected by this approach in the absence of DNA damage (Aygün et al., 2008) were detected, attesting to the reproducibility of the technique. More than 70 proteins quantified in the RNAPII IP became preferentially associated with the polymerase after DNA damage.

The well-known UV-induced interaction between RNAPII and CSB that takes place at the site of DNA damage was detected, validating the screen. As additional validations, the RPA complex and RNF168—a ubiquitin ligase involved in amplifying ubiquitin signals at sites of DNA damage (Doil et al., 2009; Marteijn et al., 2009)—were also detected. Potential components of the damage response were also uncovered. For example, the Cohesin complex interacted much more with RNAPII upon DNA damage. Cohesin has multiple functions (Dorsett and Merkenschlager, 2013), including a role in the response to UV damage in yeast (Nagao et al., 2004). The amount of Cohesin in the RNAPII IP, as measured by the intensity-based absolute quantification (iBAQ) value (reflecting absolute protein abundance) (Schwanhäusser et al., 2011), was much larger than that of RPA or CSB, for example, suggesting that Cohesin association with RNAPII increases widely; i.e., that it is not confined to actual sites of DNA damage. Several factors connected to transcript elongation, such as the RNAPII CTD-kinase CDK9, the histone H3-K36me3 methyltransferase SETD2, the PAF complex, the helicase RECQL5, and the CTD-binding proteins SCAF4 and SCAF8 were also identified as UV-induced RNAPII interactors.

We note that it remains unknown how damage-stalled RNAPII is initially recognized. Among proteins with a TFIIS-like RNAPII-binding domain (TFS2M), TCEA1 and TCEA2 (encoding TFIIS), PHF3, and DIDO1 were detected as RNAPII interactors, but only PHF3 was recruited in response to DNA damage. The PHRF1 protein was also recruited; it contains a PHD domain, which binds methylated histone H3K36, possibly put in place by the co-recruited SETD2 protein.

We also note that several proteins were lost from RNAPII upon DNA damage (Figure 2C; Table S3). For example, interactions with transcription initiation factors such as TFIIF (GTF2F), the oncoprotein MYC, mRNA capping protein CMTR1, and termination factor XRN2 were markedly reduced. Although further mechanistic studies will be required, these changes might help explain the DNA-damage-induced, global transcription shutdown observed after UV irradiation.

Chromatin Proteome

TC-NER factors such as CSA associate tightly with chromatin only upon DNA damage (Kamiuchi et al., 2002), prompting us to identify proteins that are associated with chromatin before and after DNA damage (Figure 3A; Table S4). At the same time, the chromatin proteome served as a reference proteome (input sample) for the interactomes described above.

Proteins with a markedly increased presence in chromatin after DNA damage, such as EMC8 and the dehydrogenase HIBADH were observed, irrespective of treatment with MG132. Conversely, other proteins appeared to be depleted from chromatin upon UV irradiation. Some of these might be candidates for UV-induced proteasomal degradation. More than 150 proteins were markedly lost from chromatin in response to UV irradiation in the absence of MG132, with 50 of these failing to disappear in the presence of the proteasome inhibitor. As expected, RNAPII was among the latter proteins, but other interesting factors, such as interacts with SPT6 1 (IWS1), also disappeared upon DNA damage unless the proteasome was inhibited. The abundance of its partner, SPT6 (SUPT6H), was reduced after UV treatment in the absence of MG132, but not in its presence (Figure 3A; Table S4).

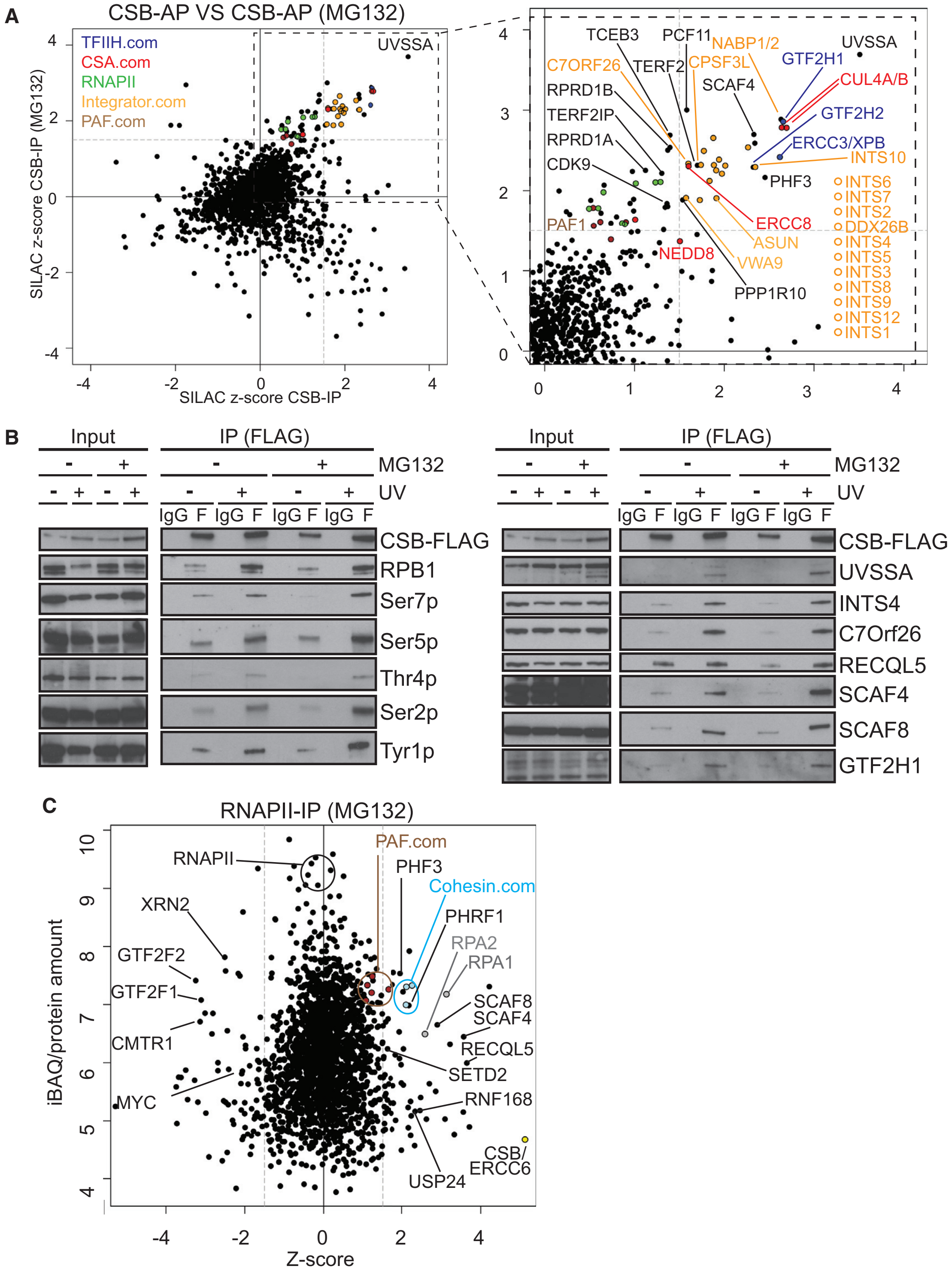

Figure 2. Effect of UV-Induced DNA Damage on the CSB and RNAPII Interactomes

(A) Left: UV-induced CSB interactome, in the presence and absence of MG132 as indicated. Right: enlargement of section indicated by box on the left. For clarity, only a few interesting proteins are indicated. Integrator subunits are labeled in yellow.

(B) Western blots of CSB-Flag immunoprecipitation. The CSB-FLAG panel is duplicated to indicate that the panel rows belong to the same experiment. Note that CSB does not seem to enrich a specific, phosphorylated form of RNAPII (left panel).

(C) The RNAPII interactome, in the presence of MG132. Some interesting proteins are indicated. Other proteins can be searched at http://www.biologic-db.org. See also Tables S1 and S2.

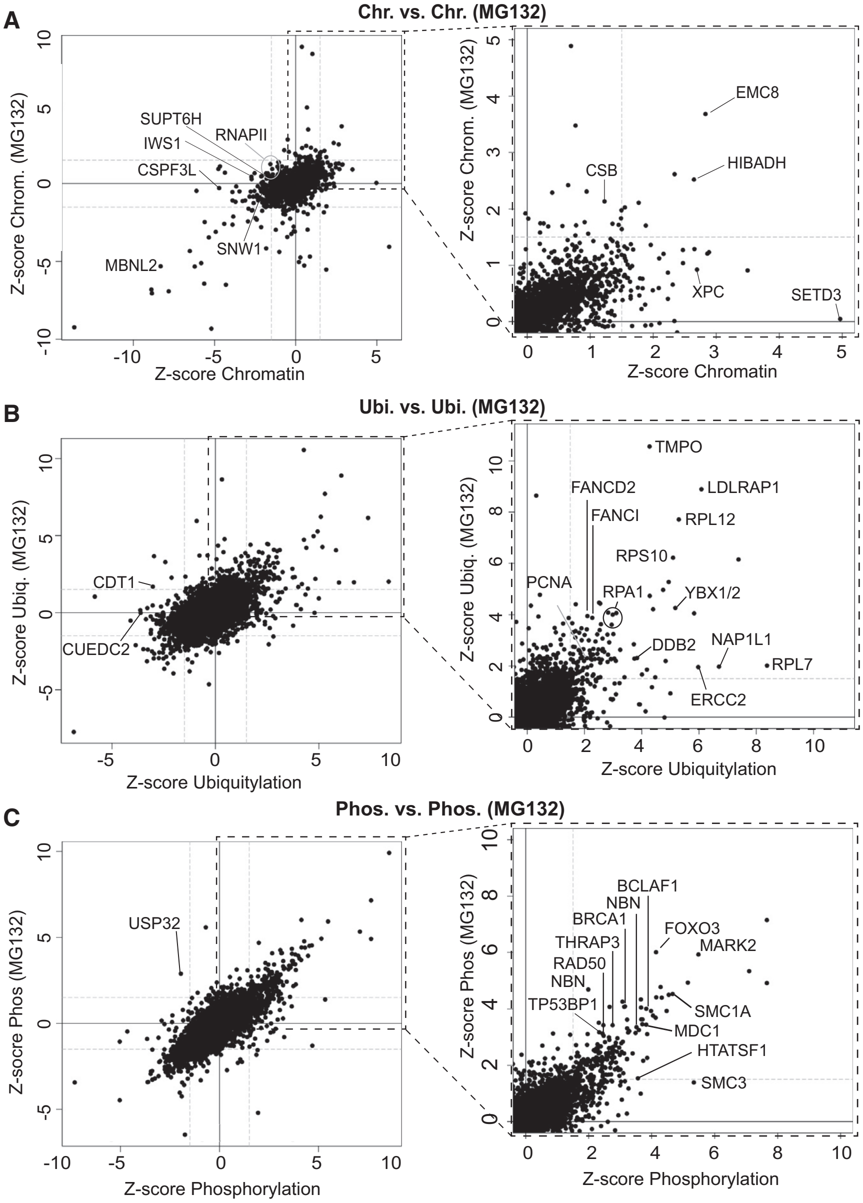

Figure 3. Effect of UV-Induced DNA Damage on the Chromatin Proteome, Ubiquitylome, and the Phosphoproteome

(A) Left: effect of UV irradiation on the chromatin proteome in the presence and absence of MG132, as indicated. Right: enlargement of section indicated by box on the left. A few proteins are indicated.

(B) As in (A), but for ubiquitylation.

(C) As in (A) and (B), but phosphorylation. Other proteins can be searched at http://www.biologic-db.org.

See also Tables S3, S4, and S5.

UV Ubiquitylome

We next used SILAC proteomics in combination with affinity purification of ubiquitin remnants to identify >10,000 ubiquitylation sites, proteome-wide. Of these, ~900 were affected by DNA damage. As a positive control, and consistent with prior work by others (Povlsen et al., 2012; Elia et al., 2015b), we detected markedly increased levels of RPA1- (K163, K167, K331), $PCNA_{K164}$-, $FANCI_{K523}$-, and $FANCD2_{K561}$ ubiquitylation upon UV treatment (Figure 3B; Table S5).

Ubiquitylation of Cohesin subunits changed markedly upon DNA damage: $RAD21_{K573}$ became ubiquitylated, while SMC1A appears to become de-ubiquitylated at several sites, further reinforcing the connection suggested by the RNAPII interactome. The YBX proteins, involved in both transcriptional and translational control (Matsumoto and Bay, 2005), became heavily ubiquitylated after UV irradiation as well. A connection between YBX proteins and the DNA damage response has not previously been reported, but the fact that elevated levels of these proteins occur in a number of human malignancies and is associated with poor prognosis and disease recurrence (Kosnopfel et al., 2014), is potentially significant in this connection. Interestingly, however, the group of proteins that appeared to have the most marked increase in site-specific ubiquitylation comprised ribosome proteins and included RPS10, RPL7, and RPL12 (Figure 3B; Table S5).

UV Phosphoproteome

We also recorded the UV-induced phosphoproteome (Figure 3C; Table S6A). Serine, threonine, and tyrosine phosphorylation sites were detected. Of these, 543 serines, 91 threonines, and 1 tyrosine (MAPK9 Y185) were markedly more phosphorylated in response to UV irradiation. As expected, damage-induced phosphorylation of H2AX (H2AFX) at serine 140 was detected (γH2AX).

By analyzing the sequence motifs that increase in phosphorylation status after UV irradiation, we found that the ATM/ATR consensus motif S/T-Q was generally enriched. In total, we detected 396 S/TQ phosphorylation sites. Forty-five of these were not described in the phosphosite plus reference database (http://www.phosphosite.org) and 14 of these increased in phosphorylation in response to UV irradiation. Lists of these sites can be found in Tables S6B and S6C.

Intriguingly, the Cohesin complex was also phosphorylated at several sites in a UV-induced manner. Nipped-B-like (NIPBL), an essential part of the Cohesin loading-complex, had a UV-induced ATM/ATR phosphorylation site as well. Moreover, a wide variety of other proteins, which have primarily been implicated in the DNA double strand break response were also phosphorylated after UV irradiation. These included ATRIP, BRCA1, CHEK1, CHEK2, CLSPN, FANCD2, MDC1, NBN, RAD50, TIPIN, TP53BP1, and XRCC4, BCLAF1, and THRAP3.

OPEN
ACCESS
CellPress

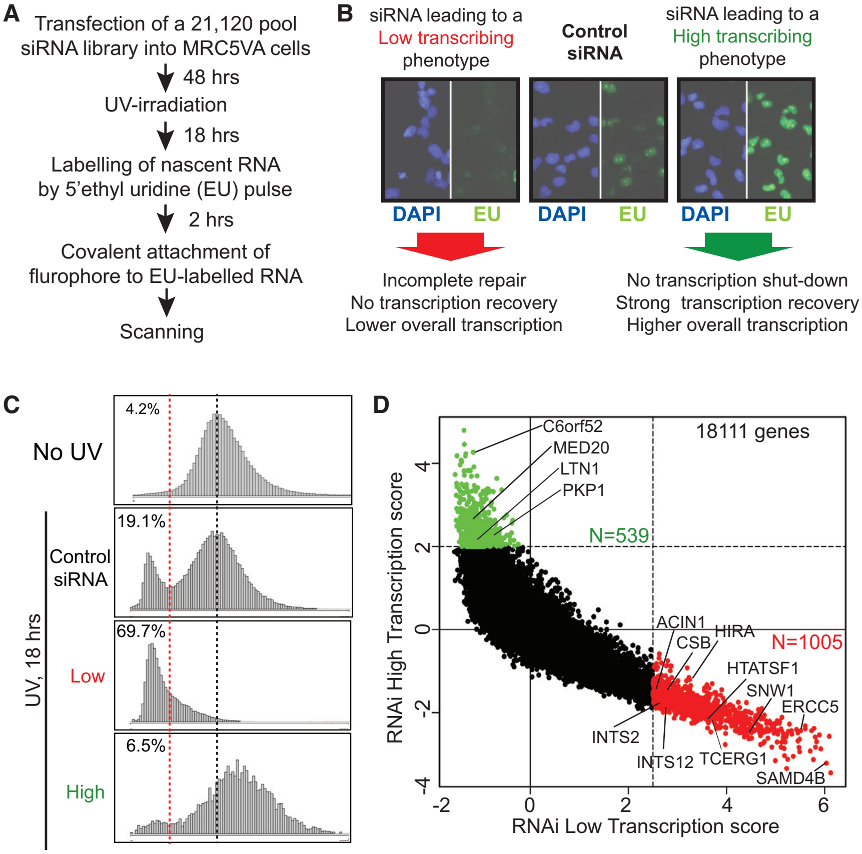

Figure 4. An siRNA Screen for Genes Affecting Transcription upon UV Irradiation

(A) The experimental approach.

(B) Typical examples of siRNAs that result in either (left) "low transcription," or (right) "high transcription," relative to the controls (middle). Different putative causes (not necessarily mutually exclusive) of the outcome are listed below arrows.

(C) Nascent transcription profiles across a cell population in the absence of UV irradiation, and in the examples from (B), used to identify siRNAs giving rise to low and high transcription, respectively. EU intensity (y axis) across the population of cells in an individual plate well (x axis) is shown.

(D) Graphical representation of the screen result. High transcribers are labeled green, and low transcribers are red. Specific genes are indicated. Other proteins can be searched at http://www.biologic-db.org.

RNAi Screen

A genome-wide small interfering RNA (siRNA) screen, surveying gene products that affect transcription after UV irradiation, complemented the proteomic screens above. Briefly, Dharmacon siRNA SMARTpools were used to induce knockdown. Upon UV irradiation, cells were allowed to recover for 18 hr before nascent transcription was measured (Figures 4A and 4B). siRNA pools targeting CSB and RNAPII, which should both decrease nascent RNA synthesis (CSB knockdown specifically so after UV irradiation), were included as positive controls, while non-targeting siRNAs, and siRNAs that are not taken up by the RNA-induced silencing (RISC) complex and thus do not lead to knockdown of any gene (RISC-free siRNA), were included as negative controls. In the absence of UV irradiation, average nascent transcription per nucleus (as measured by 5′ ethynyl uridine [EU] incorporation) followed a normal distribution (Figure 4C, No UV). However, in response to UV, a distinct population of lowly transcribing cells was clearly detectable, even after 18–20 hr (Figure 4C, Control). siRNAs giving rise to low transcription markedly increased the percentage of such cells (Figure 4C, Low). Other siRNAs resulted in a significant shift of the profile toward the right, suggesting high levels of transcription in these cells (Figure 4C, High). The results from the genome-wide screen are summarized in Figure 4D (see also Table S7). siRNAs targeting NER- or TC-NER-related gene products such as ERCC1, XAB2, HIRA, ERCC5 (XPG), TTDA, and ERCC4 (XPF) resulted in low transcription, and the known NER factors were generally significantly represented in the list of siRNA targets that reduced transcription after UV irradiation ($p = 0.01142$), validating the approach. A number of interesting factors affected transcription in this screen, including INTS2, INTS12, ACIN1, HTATSF1, STK19, SAMD4B, LARP7L, HNRNPCL1, NAE1, NOP58, PRPF31, EXOSC3, FIP1L1, MOV10, PAXIP1, ISY1, SMU1, and SNW1 (low transcribers), as well as MED20, LTN1, and PKP1 (high transcribers).

Although a significant number of genuine RNAi hits are likely missed due to RNAi-induced lethality or insufficient knockdown, the results from the functional genomics screen are particularly important as they indicate the functional significance of the proteins uncovered in the descriptive proteomic screens.

Data Integration: Individual Proteins, Networks, and Pathways

In all proteomic and genomic approaches, long lists of protein/gene hits are generated, but it can often be difficult to determine which of the "hits" are biologically meaningful. In the multiomic approach, information from the other screens can be used to significantly enrich information gained from a screen in question, greatly increasing confidence in the relevance of the result. In addition, such data integration can often provide important information about the potential molecular mechanisms behind the involvement of a given gene/protein. There are numerous examples of this in the data, but here we focus on the phosphoproteome to illustrate the point.

It was immediately apparent that some kinases that had not previously been connected to the transcription-related DNA damage response must play an important role, such as the positive transcription elongation factor b (pTEFb) complex (containing CDK9 kinase). First, we found that CDK9 itself interacts much more with both RNAPII and CSB upon DNA damage, strongly suggesting a damage-induced role in transcription or repair. Moreover, 21 known CDK9 partners and interactors featured a

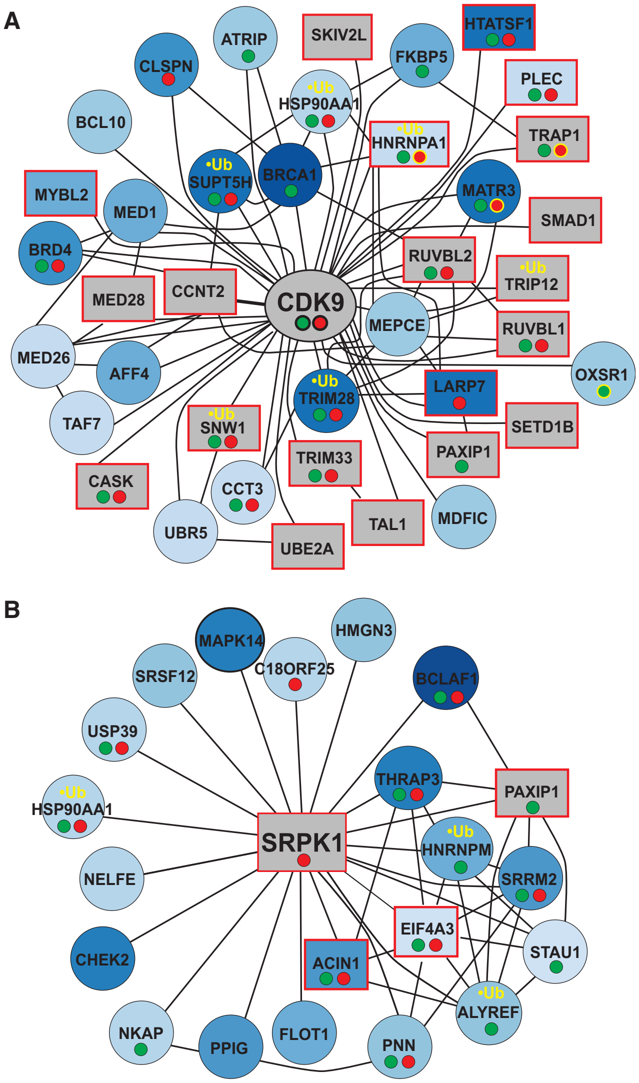

Figure 5. Enriching the Phosphoproteome with Results from the Other Screens

(A) Proteins that interact with CDK9 (pTEFb) and that become phosphorylated upon UV irradiation. Proteins are labeled increasingly blue with increasing phosphorylation. Proteins that scored in the RNAi screen, (squares with red border), interacted with RNAPII (small green spheres under name), interacted with CSB (red spheres), or became ubiquitylated upon UV irradiation (yellow "●Ub") are indicated. Examples of CSB or RNAPII interactions that increased (black circle around spheres) or decreased (yellow circle around spheres) upon UV irradiation are also specified.

(B) As in (A), but for proteins that interact with SRPK1.

UV-induced phosphorylation event (12.5%) (Figure 5A; Table S8). Among the CDK9 interactors, numerous scored in the RNAi screen and many interacted with RNAPII, CSB, or both. Importantly, one of the CDK9 cyclins, CCNT2, scored in the RNAi screen. HTATSF1, a pTEFb partner (Zhou et al., 1998), was strongly phosphorylated at several sites upon UV irradiation and scored as a low-transcriber in the RNAi screen as well. In addition, LARP7 regulates pTEFb activity by binding to and stabilizing 7SK RNA (He et al., 2008), which is in turn released from pTEFb in a protein phosphatase (PP2B/PPP3CA and PP1α/PPP1CA)-dependent manner (Chen et al., 2008). Interestingly, PPP1CA scored in the siRNA screen, and both PPP1CA and its regulatory subunit PPP1R10 interacted with RNAPII and CSB. Indeed, PPP1R10 was markedly recruited to CSB upon UV irradiation, like CDK9. PPP1R10 contains a domain, TFIIS-N, which is also found in transcription proteins such as MED26, Elongin A, IWS1, and TFIIS, raising the intriguing possibility that this domain is important for PPP1R10's proposed role in transcription and in the transcription-related DNA damage response in particular. Together, these results place PTEFb (CDK9 and its cyclin partners) at the core of the transcription-related DNA damage response.

Several well-known DNA-damage kinases were associated with a large number of increasingly phosphorylated proteins. For example, ATM kinase has 26 known interactors that were increasingly phosphorylated (22.8%), and ATR (16; 19.5%) and CHEK2 (10; 18.9%) had many as well (see Table S8). These kinases are all best known for their role in signaling double-strand breaks, reinforcing the connection to this pathway observed in several of our screens.

We also further examined potential transcription-related DNA damage-signaling kinases by focusing on those that scored in the RNAi screen and were associated with a large number of UV-induced phosphorylation events. These were SRPK1 (associated with 20 proteins showing UV-induced phosphorylation [9.5% of its interactors]), CSNK2A2 and ILK (both 15; 13.1% and 7.8%, respectively), CLK2 (13; 20.6%), and CDK8 (12; 16.4%). Forty-five other protein kinases scored in the RNAi screen, and 35 of these were associated with at least one UV-induced phosphorylation event.

A network analysis of SRPK1-associated proteins revealed that the two most highly phosphorylated SRPK1-interacting proteins are the tumor-associated genes BCLAF1 and THRAP3, both of which interact with both RNAPII and CSB (Figure 5B, blue spheres; Table S8). Another SRPK1-interacting phosphoprotein, apoptotic chromatin condensation inducer 1 (ACIN1), is particularly interesting as it also interacts with both RNAPII and CSB and scores as a low transcriber in the siRNA screen. SRPK1 has been reported as a cisplatin sensitivity factor (Schenk et al., 2001), providing an intriguing link to NER. In general, SRPK1, BCLAF1, THRAP3, and ACIN1 are associated with several networked proteins (PAXIP1, HNRNPM, SRRM2, EIF4A3, PNN, ALYREF, and STAU1) that also scored in many of our screens, including the RNAi screen (Figure 5B). Together, these data suggest a previously unrecognized role for the splicing-related kinase SRPK1 and its network partners in the transcription-related DNA damage response. Numerous other examples of affected pathways can be analyzed by the use of bioLOGIC.

Data Integration—Intersecting and Weighting Individual Screens

Finding new factors with a role in the transcription-related DNA damage response was the main initial motivation for this work. To uncover such factors, we used several different approaches to create ranked score lists. Initially, we simply awarded a point to each gene/protein scoring above the *Z* score threshold in an individual experiment. This yielded a distribution of scores,

OPEN ACCESS
CellPress

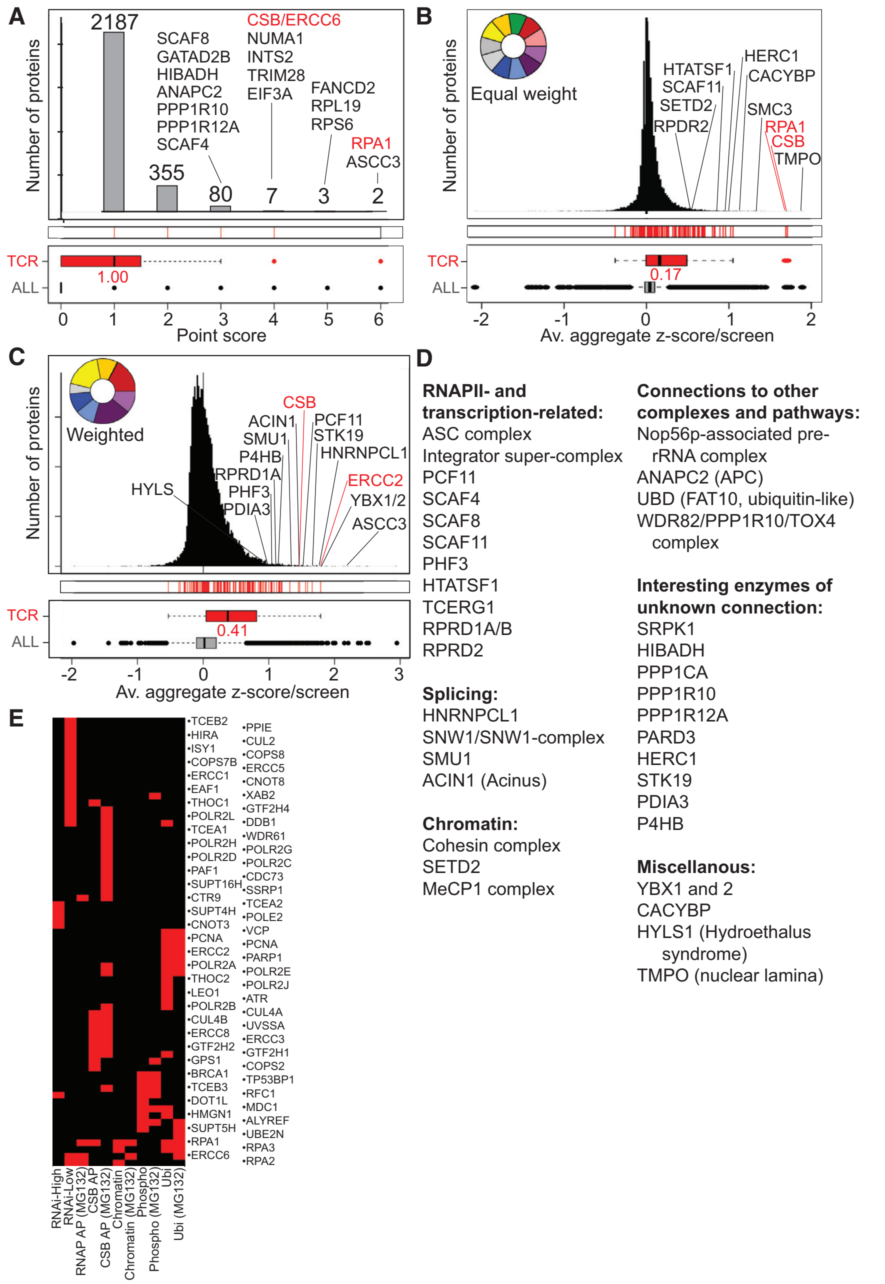

Figure 6. Proteins and Processes that Score Highly in the Multiomic Screening Approach

(A) Bar graph showing the number of proteins that scored above the Z score threshold in one or more screen.

(B) Distribution of hits using aggregated Z scores.

(C) As in (B), but with TC-NER score weighting. In (B) and (C), the colored wheels indicate relative weighting of scores from individual screens (see Figure S1A). Names and dots in red are examples from the TC-NER training category ("TCR"). Please note that, for clarity, even if a candidate scores highly in several scoring schemes, it is typically only indicated once.

(D) Biased list of interesting proteins and protein complexes that scored highly.

(E) Proteins from the TC-NER training category that scored above the Z score threshold (indicated by red bar) in screens across the multiomic approach (see Figure S1B).

See also Figures S2, S3, and S4.

from almost 2,200 proteins scoring in one screen, to only two genes, RPA1 and ASCC3, scoring in six (Figure 6A; Table S9). Realizing that setting arbitrary Z score threshold for inclusion might not be ideal, we also ranked candidates based on aggregate Z scores (Figure 6B; Table S9). None of these approaches take into account the possibility that some screens might be much better at uncovering relevant factors than others. To address this, we created a comprehensive list of "transcription-repair coupling factors" (Table S10), based on an authoritative recent review (Gaillard and Aguilera, 2013) and our own knowledge of the published literature. This category was then used as a "training category" to benchmark the individual experiments for information value regarding the known TC-NER factors. The underlying assumption is that unknown factors in the transcription-related DNA damage response will often (although obviously not invariably) follow the same pattern in the data as the known TC-NER factors. As expected, the screens had varying abilities to capture proteins from this training category, with the CSB interactome, damage-induced ubiquitylation, and RNAi low transcription particularly effective in uncovering such factors (Figure S1A). Weighting the individual screening experiments according to their performance in this respect and applying it to score all proteins increased the median score of known TC-NER protein from 0.17 to 0.41 (Figure 6C; Table S9).

It is important to emphasize that there is no single "correct way" of compiling score lists. However, if a factor scores highly no matter which method is used, this obviously increases confidence. Nevertheless, even factors that only scored highly by one or two methods might still be interesting and included with high confidence after an assessment of the underlying core data. A non-exhaustive list of high-scoring proteins, which we thought to be of particular interest and of high confidence, is shown in Figure 6D.

Next, we determined which cellular pathways are enriched in the list of high-scoring proteins (Table S11). For simplicity, this analysis was performed with the data obtained by weighted scoring (Figure 6C), but similar results were achieved using the other scoring approaches (data not shown). Gratifyingly, several pathways related to "nucleotide excision repair" were enriched (adjusted p values of 2.04×10^{-5} to 9.78×10^{-3}), but other

pathways such as "mRNA translation and ribosomes," "virus lifecycle, -transcription, and -translation," and "mRNA splicing" were highly enriched in our data as well. We also noted a broad-based connection to "double-strand break repair," which supports the idea that the response to UV-induced DNA damage is both multi-pronged and extensive. Importantly, most of the pathways were not highly enriched in the individual experiments (Table S11), consistent with the idea that the triggered pathways can be detected with higher confidence when taking several independent experimental approaches into consideration.

Gene set enrichment analysis (GSEA), originally developed for interpreting gene expression data (Subramanian et al., 2005), illustrates the enrichment of NER factors in our datasets: the proteins uncovered from this category (no less than 66 out of the 125 proteins in it) scored widely across the screens (Figure 6E; see also Figures S1B and S2). This was in contrast to some other gene ontology categories showing high overall enrichment, such as "ribosome-related" categories, where enrichment was based primarily on very high scores in the ubiquitylation screens (Figure S3).

We also queried the screen results against the Corum database of protein complexes (http://mips.helmholtz-muenchen.de/genre/proj/corum/). Indeed, the importance of a single subunit of a protein complex scoring in a single screen might be considered doubtful, but if several subunits score in several screens, then the involvement of that complex can be stated with greater confidence. A list of Corum complexes and their association with the transcription-related DNA damage response can be found in Table S12. Besides detecting repair-associated complexes such as the ubiquitin ligase complex containing CSA, this analysis uncovered protein complexes that have not previously been connected to the UV damage response, such as Integrator, MeCP1 histone deacetylase complex, and several others.

Enrichment analysis of the results from multiomic screening thus enables discovery of new systems-wide connections in the DNA damage response.

Integrating with Other Databases

To enable easy interrogation of the screen results, we developed a new database interface, named bioLOGIC (http://www.biologic-db.org). Besides allowing visualization and superimposition of results from different selected screens, bioLOGIC also enables easy integration with public databases, such as those detailing common cancer drivers, or prior DNA damage-focused screens. Furthermore, it allows rapid assessment of other information about a factor of interest, via one-click links to information in databases such as UniProt, CORUM, BioGrid, etc.

Although the multiomic approach does not integrate all publicly available information and cannot substitute for expert knowledge, it provides a powerful way of ranking genes/proteins by their strength of candidacy of being involved in the transcription-related DNA damage response. For example, the highest scoring gene/protein in the multiomic screening approach, ASCC3, was found to interact with both RNAPII and CSB, which was independently confirmed by IP-western blotting (Figure S4), pointing to a direct effect on transcription and/or repair. ASCC3 also becomes highly ubiquitylated and phosphorylated in response to UV irradiation, suggesting regulation via post-translational modification. Other results showing an involvement of ASCC3 in the transcription-related DNA damage response will be described separately (L.W., A.S., J.S., S.B., G.P.K., M.H., M. Saponaro, P. East, R. Mitter, A. Lobley, J. Walker, and B. Spencer-Dene, unpublished data), but as a further illustration of the power of the multiomic approach, we describe the initial findings on serine-threonine kinase 19 (STK19).

STK19, a poorly studied protein, would be unlikely to be pursued based on the results from any of the individual screens, but it scored in the top 25 of hits ranked by the weighted scoring approach (Figure 6C; Table S9). Specifically, STK19 interacts with CSB after DNA damage, and its knockdown affects transcription recovery after DNA damage. Using bioLOGIC to cross-reference all high-scoring proteins with cancer databases made it clear that STK19 is potentially of great interest. Indeed, it is mutated in melanoma (Hodis et al., 2012) and listed among the Broad Institute cancer driver genes (Lawrence et al., 2014), yet its function has remained undetermined.

STK19 Is Important for the Transcription-Related DNA Damage Response

To further characterize STK19, we investigated the effect of its knockdown on global transcription both in the presence and absence of DNA damage. STK19 knockdown had little effect on transcription in the absence of UV irradiation or on the global shutdown of transcription immediately after DNA damage (2 hr) (Figure 7A, left and middle panels). However, cells depleted for STK19 were clearly deficient in the recovery of transcription after DNA damage (Figures 7A, right panels, and 7B), similar to what is observed in CSB knockdown cells (see Figure S5).

To investigate how this correlated with cell viability after DNA damage, we also performed a clonogenic UV sensitivity assay (Figure 7C). Gratifyingly, cells lacking STK19 were indeed UV-sensitive, and this held true with any of the individual siRNAs from the Dharmacon pool that knocked down STK19 (Figures 7D and 7E). We also investigated whether STK19 might work at least partly via being recruited to DNA damage. For this purpose, GFP-tagged STK19 was expressed in HEK293 cells, and the localization of the protein tested after local laser-induced DNA damage. STK19, indeed, accumulated in areas of such DNA damage (Figure 7F).

These data expose the melanoma gene STK19 as a factor in the transcription-related DNA damage response.

DISCUSSION

During evolution, cells have developed sophisticated responses to genomic insult, ranging from delaying progression through the cell cycle to first repairing DNA damage where it matters most, namely in active genes. In this report, we describe a systems approach to discovering new DNA damage response factors, with particular emphasis on transcription.

The advent of sensitive techniques for genome- and proteome-wide analysis now enables the screening of mammalian

OPEN
ACCESS
CellPress

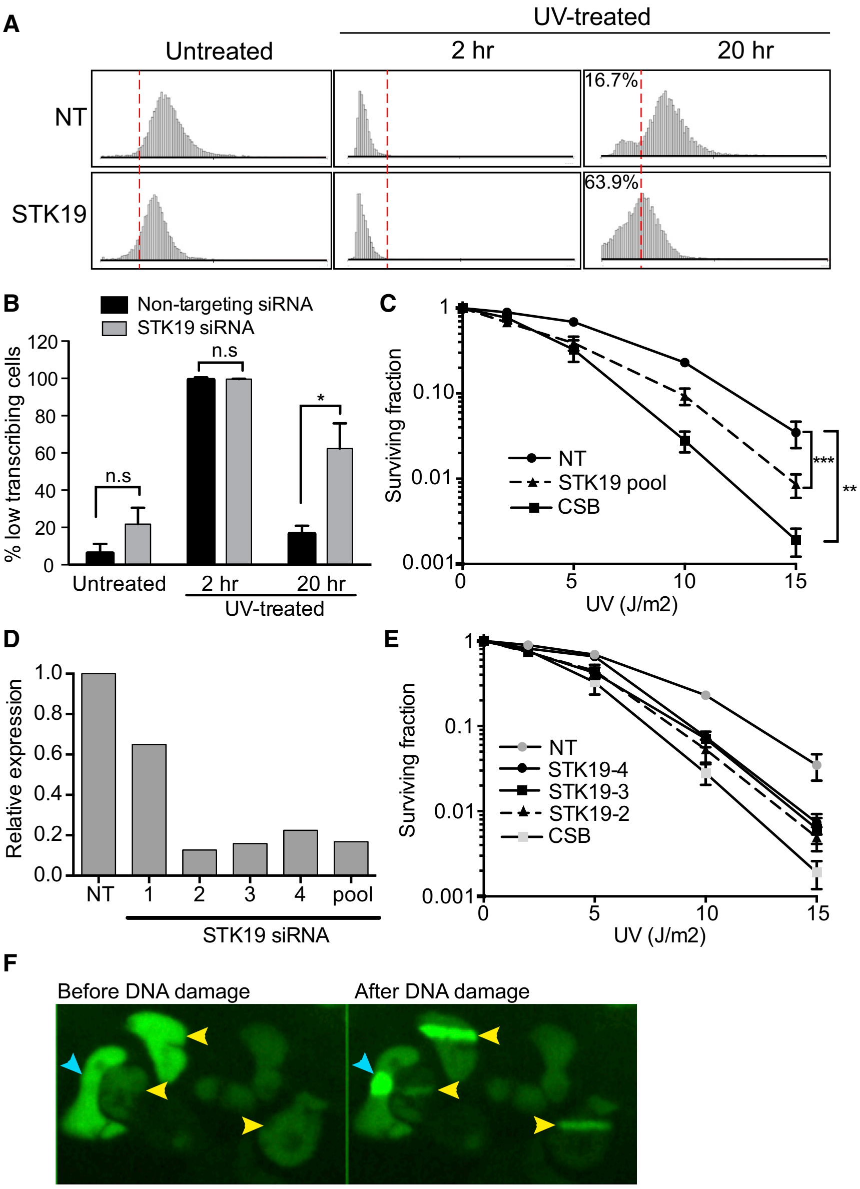

Figure 7. Involvement of STK19 in the Transcription-Related DNA Damage Response

(A and B) Lack of normal transcription recovery in cells lacking STK19.

(C–E) Cells lacking STK19 are UV-sensitive. The individual siRNAs that knock down STK19 (D) also give rise to UV sensitivity (E). The result of CSB knockdown is shown for comparison. NT, non-targeting siRNA.

(F) Recruitment of GFP-tagged STK19 to DNA damage induced by laser micro-irradiation in a diffraction-limited spot (blue arrows) or stripe (yellow arrows). Cells were imaged immediately before and 2 hr after micro-irradiation.

See also Figures S4 and S5 and the Supplemental Experimental Procedures for details.

cells either genetically or biochemically. The enormous amount of data from such screens is, however, typically accompanied by a fundamental problem—a very low signal-to-noise ratio. Even though experimental variation can sometimes be reduced by employing a sufficient number of replicates (typically at very substantial effort and cost), this does not eliminate principal blind spots in individual experimental techniques. In the multiomic approach described here, several independent approaches are used to characterize the same cellular response pathway. As it explores the same process from different angles, it might be viewed as the biological screening equivalent of the statistical chain rule (or general product rule) of probability: it places less emphasis on hits from any individual screen and instead focuses primarily on factors and pathways that score in several screens. Its primary aim is to discover new pathways/factors, and the approach should thus be distinguished from "cataloging approaches" where detailed information about one particular aspect of a process/reaction is obtained. While screens cataloguing phosphorylation, ubiquitylation, and transfer to chromatin in response to UV-mediated DNA damage have previously been performed (although in other cell lines and under other conditions; e.g., see Chou et al., 2010; Povlsen et al., 2012; Elia et al., 2015a), screens to map damage-induced RNAPII- and CSB-interactors have not previously been reported and neither have genome-wide screens for transcription recovery after UV irradiation. In any case, an integration of multiple screen results such as that described here is best based on screens performed under the same conditions and in the same cell lines. It is crucial to emphasize that performing several screens side-by-side also often bypasses the pressing need for independent confirmation that typifies single-screen approaches; the confirmation for a hit in, for example, the siRNA screen is thus provided when the same factor also scores as an interactor of RNAPII and/or CSB, and/or by transferring to chromatin, and/or becoming ubiquitylated or phosphorylated upon DNA damage. As a consequence, easy cross-referencing between screen results and with other published screens is important when attempting to make sense of datasets that are, by their nature, incomplete. For this and other purposes, a searchable web interface, bioLOGIC, was developed.

bioLOGIC

Biological datasets are becoming ever more complex so it is of vital importance to enable quick and intuitive access. We therefore put great emphasis on an interactive database, named bioLOGIC (http://www.biologic-db.org), which makes it straightforward to retrieve data on any individual human gene or protein,

whether it has scored in our screens or not ("bioLOGIC Data"). Importantly, bioLOGIC allows one-click access to basic information about individual candidates in different public databases, as well as immediate access to information about the protein's functional domains and about the protein complexes it belongs to. It is possible, for example, to directly learn how other subunits of the same complex score in the screens ("Category members"). Finally, bioLOGIC "Categoryview" makes it possible to quickly sample, for example, how all proteins within a particular gene ontology category scored in the screens. The bioLOGIC interface thus permits quick judgment of the relevance and importance in the DNA damage response of any protein or process of interest.

Pathways, Processes, and Complexes

At the systems level, the multiomic approach and bioLOGIC provide a birds-eye view of the cellular responses triggered by UV irradiation. As expected, DNA/chromatin- and RNA-related processes dominate the list, including processes such as mRNA splicing, RNAPII transcript elongation, chromosome maintenance, and DNA repair, and protein complexes such as Spliceosome, PAF complex, and MeCP1, for example.

A multi-level overlap between genome instability and mRNA splicing has become apparent over the last few years (Lenzken et al., 2013; Chan et al., 2014), and this connection is obvious in our data as well. Interestingly, our data also corroborate anecdotal evidence for an overlap between the response to UV irradiation and double-strand DNA breaks. This overlap might be based on a re-use of response proteins and signaling pathways for different kinds of DNA damage. However, it is equally possible that transcription-impeding damage caused by UV irradiation results in double-strand breaks more frequently than previously assumed, for example through the formation of R loops and their faulty processing, as suggested by Sollier and Cimprich (2015). Finally, it is not impossible that the doses of UV irradiation used in our study are high enough to directly cause some double-strand breaks or inter-strand DNA crosslinks, which could also help explain some of the observations.

It is worth noting that DNA damage response pathways, such as the ATM pathway, can also be activated by replication stress, which occurs as a consequence of UV-induced DNA damage at later time points (Zeman and Cimprich, 2014). Although we believe that replication stress is not a major factor in our results, a follow-up study specifically targeted at this pathway would be required to tease apart the responses to DNA damage and replication stress.

Besides the systems level overlaps described above, unexpected connections were also uncovered. The high scores of ribosomal subunits is a particularly striking example. This score is at least partly due to a dramatic increase in phosphorylation, and especially ubiquitylation, at one or more sites of a very large number of ribosomal proteins in response to UV irradiation. However, ribosomal subunit genes such as RPS27A, RPL19, and RPS6 also scored in the RNAi screen. Translation initiation factor EIF3A scored in this screen as well and is ubiquitylated and interacts more strongly with RNAPII upon UV irradiation. Intriguingly, LTN1 (Listerin), a ubiquitin ligase and component of the Ribosome Quality Control complex (Wolff et al., 2014), also scored in the siRNA screen. We noted with interest that ribosomal proteins, such as RPS6, RPS9, and RPS15A, previously also scored in a genomic screen performed by the Paulsen et al. (2009) laboratory for genes whose knockdown result in elevated γH2AX levels in response to ionizing radiation. Together, these intriguing results point to an unexplored connection between translation and the transcription-related DNA damage response that merits further investigation.

Intriguing connections to viral infection, interferon signaling, and the immune system are also worth mentioning. The interferon system is a powerful antiviral response capable of controlling virus infections in the absence of adaptive immunity (Randall and Goodbourn, 2008). The connection to interferon signaling was mostly uncovered via the RNAi screen. First, DNA-pattern receptors of the innate immune response scored in the RNAi screen (Toll-like receptor 2 (TLR2, TLR7, and TLR9). Further downstream in this cascade, components of the NF-kappa B pathway, such as CHUK, ERC1, NFKBIB, TICAM1, and NR2C2 (also known as TAK1) scored as well. Other components of the innate immune response, such as TRIM56 (linked to TLRs), also scored. The connection to virus biology is even more wide-ranging, with cellular proteins linked to HIV-Tat scoring highly in almost all screens. Again, the mechanism and significance of these results remain to be established, but we note that our own gene expression data (not shown), as well as those of others (Zaidi et al., 2011; Shen et al., 2015), also suggest an overlap between interferon signaling and the UV damage response. It is an intriguing possibility that the sophisticated and complex response of higher cells to viruses and infection might have evolved from and/or adopted aspects of a more ancient DNA damage response.

Individual Factors and Complexes

A large number of individual proteins and protein complexes scored highly across our screens. Given our interest in transcription and transcript elongation in particular, we are especially interested in the unexplored role in the DNA damage response of factors such as ASCC3 (L.W., A.S., J.S., S.B., G.P.K., M.H., M. Saponaro, P. East, R. Mitter, A. Lobley, J. Walker, and B. Spencer-Dene, unpublished data), the SCAF proteins, PCF11, PHF3, and Integrator complex. Interestingly, our experiments point to the existence of a large Integrator super-complex, including ASUN, C7ORF26, DDX26B, and VWA9/C15ORF44, as well as NABP1, which might well be a specialized form of Integrator for the DNA damage response. Indeed, Integrator super-complex not only interacted with CSB upon DNA damage, but subunits such as INTS2 and INTS12 also scored in the functional RNAi screen, while others changed their level of post-translational modification in response to UV irradiation. Similarly, the SCAF proteins, PCF11 and PHF3, also scored in several of our screens, while PCF11 and Integrator subunits also previously scored in an siRNA screen for increased damage signaling (Paulsen et al., 2009), all in all providing a solid starting point for further detailed functional analysis of their physiological role. In general, an involvement in the DNA damage response of these basal mRNA processing/termination factors might potentially help explain the dramatic downregulation of transcription occurring upon DNA damage. However, the role in the DNA damage

response of other high-scoring proteins with an often poorly understood role in transcription, such as HTATSF1, TCERG1, RPRD1A/B, RPRD2, and SND1 certainly also merits further study.

Obviously, the ultimate goal of any screening approach is to uncover new factors without prior connections to the process of interest. We believe that the multiomic approach achieved this goal; numerous proteins of unknown function, including several enzymes, were uncovered that are connected to the transcription-related DNA damage response. As an example, we followed up on two proteins, ASCC3 and STK19. Our data on ASCC3 will be described elsewhere (L.W., A.S., J.S., S.B., G.P.K., M.H., M. Saponaro, P. East, R. Mitter, A. Lobley, J. Walker, and B. Spencer-Dene, unpublished data). In the case of STK19, there is some evidence that it is a kinase (Gomez-Escobar et al., 1998), but no cellular function had been assigned to the protein. Our data now place this extremely poorly studied protein in the DNA-damage response. Indeed, the multiomic analysis showed that STK19 interaction with CSB increases dramatically after DNA damage in the presence of MG132. Moreover, in the absence of STK19, transcription fails to recover upon UV irradiation. Crucially, our follow-up experiments showed that STK19 deficiency also results in UV sensitivity, and the protein is recruited to sites of DNA damage. STK19 was previously identified in two cancer genomics studies of genes that are frequently mutated in melanoma patients (Hodis et al., 2012; Lawrence et al., 2014), making its role in the transcription-related DNA damage response particularly exciting. Uncovering the precise role of STK19 in the DNA damage response is an important future goal.

In conclusion, by investigating a complex cellular process from a number of distinct angles, the data presented here provides an unprecedented systems level view at the transcription-related DNA damage and at the same time uncovers numerous factors involved in it. The detailed study of the many connections revealed will be a major undertaking.

EXPERIMENTAL PROCEDURES

Cell Lines

HEK293 cell lines expressing FLAG-tagged proteins were used for proteomic analysis. Cells were cultured in light or heavy SILAC medium for at least seven generations, UV-irradiated, and allowed to recover at 37°C for 3 hr. Micro-irradiation and imaging was performed with a PerkinElmer UltraVIEW VoX spinning disk microscope.

Extract Preparation

Cells were lysed by dounce homogenization and nuclei pelleted by centrifugation. Following nucleoplasmic extraction, the chromatin pellet was treated with Benzonase. FLAG-M2 beads (Sigma-Aldrich) were employed for affinity purification. Elution was with 3xFLAG peptide. Antibodies were purchased from Bethyl Laboratories, Abcam, and Santa Cruz Biotechnology.

Proteomics

Anti-GlyGly antibody (Cell Signaling Technology) was used for ubiquitin-remnant profiling (Xu et al., 2010), with modifications to previous procedures. Phosphopeptide enrichment was performed using TiO_2 beads (Titansphere, 5 μm, GL Sciences). Upon liquid chromatography-tandem mass spectrometry (LC-MS/MS) analysis, raw mass spectrometry data were analyzed using MaxQuant. Parent ion and tandem mass spectra were searched against the UniprotKB *Homo sapiens* database.

RNAi Screen

The siRNA screen was performed in MRC5VA cells with the Dharmacon Human siGENOME library. Plates were exposed to short-wavelength UV (UV-C) light and then incubated for a further 18 hr before labeling nascent RNA with 5′-ethynyl uridine. Automated image acquisition was performed (Cellomics Array Scan VTI) using a 10× objective. Image analysis was performed using HCS Studio (Thermo Scientific).

For further details, please see the Supplemental Experimental Procedures.

SUPPLEMENTAL INFORMATION

Supplemental Information includes Supplemental Experimental Procedures, five figures, and twelve tables and can be found with this article online at http://dx.doi.org/10.1016/j.celrep.2016.04.047.

AUTHOR CONTRIBUTIONS

S.B. performed proteomic experiments (except for the C7ORF26 interactome, which was analyzed by O.A.). V.E. and B.S. were responsible for mass spectrometry analysis. L.W. performed the functional genomics screen, with help from I.G., R.E.S., R.I., and M.H. M.R., J.C.W., J.W.C., and R.C.C. were responsible for the experiments with STK19. S.B., G.P.K., and A.S. performed bioinformatics analysis. J.Q.S. supervised the work and wrote the paper, with input from all authors.

ACKNOWLEDGMENTS

This work was supported by the Francis Crick Institute (grant FCI01), which receives its core funding from Cancer Research UK, the UK Medical Research Council, and the Wellcome Trust. The work was also supported by grants from the European Research Council, and Worldwide Cancer Research (formerly known as Association for International Cancer Research to J.Q.S.). We thank the Francis Crick institute's Cell Service facility for assistance and Barbara Dirac Svejstrup and other members of the J.Q.S. lab for comments on the manuscript. We also thank the members of the Bioinformatics and Biostatistics Laboratory for helpful discussions and Professor Dirk Eick, University of Munich for the kind gift of RNAPII CTD antibodies.

Received: November 24, 2015
Revised: February 25, 2016
Accepted: April 10, 2016
Published: May 12, 2016

REFERENCES

Anindya, R., Mari, P.O., Kristensen, U., Kool, H., Giglia-Mari, G., Mullenders, L.H., Fousteri, M., Vermeulen, W., Egly, J.M., and Svejstrup, J.Q. (2010). A ubiquitin-binding domain in Cockayne syndrome B required for transcription-coupled nucleotide excision repair. Mol. Cell *38*, 637–648.

Aygün, O., Svejstrup, J., and Liu, Y. (2008). A RECQ5-RNA polymerase II association identified by targeted proteomic analysis of human chromatin. Proc. Natl. Acad. Sci. USA *105*, 8580–8584.

Baillat, D., and Wagner, E.J. (2015). Integrator: surprisingly diverse functions in gene expression. Trends Biochem. Sci. *40*, 257–264.

Baillat, D., Hakimi, M.A., Näär, A.M., Shilatifard, A., Cooch, N., and Shiekhattar, R. (2005). Integrator, a multiprotein mediator of small nuclear RNA processing, associates with the C-terminal repeat of RNA polymerase II. Cell *123*, 265–276.

Bounaix Morand du Puch, C., Barbier, E., Kraut, A., Couté, Y., Fuchs, J., Buhot, A., Livache, T., Sève, M., Favier, A., Douki, T., et al. (2011). TOX4 and its binding partners recognize DNA adducts generated by platinum anticancer drugs. Arch. Biochem. Biophys. *507*, 296–303.

Bradshaw, P.S., Stavropoulos, D.J., and Meyn, M.S. (2005). Human telomeric protein TRF2 associates with genomic double-strand breaks as an early response to DNA damage. Nat. Genet. *37*, 193–197.

Chan, Y.A., Hieter, P., and Stirling, P.C. (2014). Mechanisms of genome instability induced by RNA-processing defects. Trends Genet. *30*, 245–253.

Chen, R., Liu, M., Li, H., Xue, Y., Ramey, W.N., He, N., Ai, N., Luo, H., Zhu, Y., Zhou, N., and Zhou, Q. (2008). PP2B and PP1alpha cooperatively disrupt 7SK snRNP to release P-TEFb for transcription in response to Ca2+ signaling. Genes Dev. *22*, 1356–1368.

Chou, D.M., Adamson, B., Dephoure, N.E., Tan, X., Nottke, A.C., Hurov, K.E., Gygi, S.P., Colaiácovo, M.P., and Elledge, S.J. (2010). A chromatin localization screen reveals poly (ADP ribose)-regulated recruitment of the repressive polycomb and NuRD complexes to sites of DNA damage. Proc. Natl. Acad. Sci. USA *107*, 18475–18480.

Christiansen, M., Stevnsner, T., Modin, C., Martensen, P.M., Brosh, R.M., Jr., and Bohr, V.A. (2003). Functional consequences of mutations in the conserved SF2 motifs and post-translational phosphorylation of the CSB protein. Nucleic Acids Res. *31*, 963–973.

Doil, C., Mailand, N., Bekker-Jensen, S., Menard, P., Larsen, D.H., Pepperkok, R., Ellenberg, J., Panier, S., Durocher, D., Bartek, J., et al. (2009). RNF168 binds and amplifies ubiquitin conjugates on damaged chromosomes to allow accumulation of repair proteins. Cell *136*, 435–446.

Dorsett, D., and Merkenschlager, M. (2013). Cohesin at active genes: a unifying theme for cohesin and gene expression from model organisms to humans. Curr. Opin. Cell Biol. *25*, 327–333.

Elia, A.E., Boardman, A.P., Wang, D.C., Huttlin, E.L., Everley, R.A., Dephoure, N., Zhou, C., Koren, I., Gygi, S.P., and Elledge, S.J. (2015a). Quantitative proteomic atlas of ubiquitination and acetylation in the DNA damage response. Mol. Cell *59*, 867–881.

Elia, A.E., Wang, D.C., Willis, N.A., Boardman, A.P., Hajdu, I., Adeyemi, R.O., Lowry, E., Gygi, S.P., Scully, R., and Elledge, S.J. (2015b). RFWD3-Dependent Ubiquitination of RPA Regulates Repair at Stalled Replication Forks. Mol. Cell *60*, 280–293.

Fousteri, M., Vermeulen, W., van Zeeland, A.A., and Mullenders, L.H. (2006). Cockayne syndrome A and B proteins differentially regulate recruitment of chromatin remodeling and repair factors to stalled RNA polymerase II in vivo. Mol. Cell *23*, 471–482.

Gaillard, H., and Aguilera, A. (2013). Transcription coupled repair at the interface between transcription elongation and mRNP biogenesis. Biochim. Biophys. Acta *1829*, 141–150.

Gomez-Escobar, N., Chou, C.F., Lin, W.W., Hsieh, S.L., and Campbell, R.D. (1998). The G11 gene located in the major histocompatibility complex encodes a novel nuclear serine/threonine protein kinase. J. Biol. Chem. *273*, 30954–30960.

Groisman, R., Polanowska, J., Kuraoka, I., Sawada, J., Saijo, M., Drapkin, R., Kisselev, A.F., Tanaka, K., and Nakatani, Y. (2003). The ubiquitin ligase activity in the DDB2 and CSA complexes is differentially regulated by the COP9 signalosome in response to DNA damage. Cell *113*, 357–367.

Groisman, R., Kuraoka, I., Chevallier, O., Gaye, N., Magnaldo, T., Tanaka, K., Kisselev, A.F., Harel-Bellan, A., and Nakatani, Y. (2006). CSA-dependent degradation of CSB by the ubiquitin-proteasome pathway establishes a link between complementation factors of the Cockayne syndrome. Genes Dev. *20*, 1429–1434.

He, N., Jahchan, N.S., Hong, E., Li, Q., Bayfield, M.A., Maraia, R.J., Luo, K., and Zhou, Q. (2008). A La-related protein modulates 7SK snRNP integrity to suppress P-TEFb-dependent transcriptional elongation and tumorigenesis. Mol. Cell *29*, 588–599.

Hodis, E., Watson, I.R., Kryukov, G.V., Arold, S.T., Imielinski, M., Theurillat, J.P., Nickerson, E., Auclair, D., Li, L., Place, C., et al. (2012). A landscape of driver mutations in melanoma. Cell *150*, 251–263.

Kamiuchi, S., Saijo, M., Citterio, E., de Jager, M., Hoeijmakers, J.H., and Tanaka, K. (2002). Translocation of Cockayne syndrome group A protein to the nuclear matrix: possible relevance to transcription-coupled DNA repair. Proc. Natl. Acad. Sci. USA *99*, 201–206.

Kosnopfel, C., Sinnberg, T., and Schittek, B. (2014). Y-box binding protein 1–a prognostic marker and target in tumour therapy. Eur. J. Cell Biol. *93*, 61–70.

Lawrence, M.S., Stojanov, P., Mermel, C.H., Robinson, J.T., Garraway, L.A., Golub, T.R., Meyerson, M., Gabriel, S.B., Lander, E.S., and Getz, G. (2014). Discovery and saturation analysis of cancer genes across 21 tumour types. Nature *505*, 495–501.

Lenzken, S.C., Loffreda, A., and Barabino, S.M. (2013). RNA splicing: a new player in the DNA damage response. Int. J. Cell Biol. *2013*, 153634.

Malovannaya, A., Li, Y., Bulynko, Y., Jung, S.Y., Wang, Y., Lanz, R.B., O'Malley, B.W., and Qin, J. (2010). Streamlined analysis schema for high-throughput identification of endogenous protein complexes. Proc. Natl. Acad. Sci. USA *107*, 2431–2436.

Marteijn, J.A., Bekker-Jensen, S., Mailand, N., Lans, H., Schwertman, P., Gourdin, A.M., Dantuma, N.P., Lukas, J., and Vermeulen, W. (2009). Nucleotide excision repair-induced H2A ubiquitination is dependent on MDC1 and RNF8 and reveals a universal DNA damage response. J. Cell Biol. *186*, 835–847.

Matsumoto, K., and Bay, B.H. (2005). Significance of the Y-box proteins in human cancers. J. Mol. Genet. Med. *1*, 11–17.

Mayne, L.V., and Lehmann, A.R. (1982). Failure of RNA synthesis to recover after UV irradiation: an early defect in cells from individuals with Cockayne's syndrome and xeroderma pigmentosum. Cancer Res. *42*, 1473–1478.

Morales, J.C., Richard, P., Rommel, A., Fattah, F.J., Motea, E.A., Patidar, P.L., Xiao, L., Leskov, K., Wu, S.Y., Hittelman, W.N., et al. (2014). Kub5-Hera, the human Rtt103 homolog, plays dual functional roles in transcription termination and DNA repair. Nucleic Acids Res. *42*, 4996–5006.

Nagao, K., Adachi, Y., and Yanagida, M. (2004). Separase-mediated cleavage of cohesin at interphase is required for DNA repair. Nature *430*, 1044–1048.

Ni, Z., Xu, C., Guo, X., Hunter, G.O., Kuznetsova, O.V., Tempel, W., Marcon, E., Zhong, G., Guo, H., Kuo, W.H., et al. (2014). RPRD1A and RPRD1B are human RNA polymerase II C-terminal domain scaffolds for Ser5 dephosphorylation. Nat. Struct. Mol. Biol. *21*, 686–695.

Ong, S.E., Blagoev, B., Kratchmarova, I., Kristensen, D.B., Steen, H., Pandey, A., and Mann, M. (2002). Stable isotope labeling by amino acids in cell culture, SILAC, as a simple and accurate approach to expression proteomics. Mol. Cell. Proteomics *1*, 376–386.

Paulsen, R.D., Soni, D.V., Wollman, R., Hahn, A.T., Yee, M.C., Guan, A., Hesley, J.A., Miller, S.C., Cromwell, E.F., Solow-Cordero, D.E., et al. (2009). A genome-wide siRNA screen reveals diverse cellular processes and pathways that mediate genome stability. Mol. Cell *35*, 228–239.

Povlsen, L.K., Beli, P., Wagner, S.A., Poulsen, S.L., Sylvestersen, K.B., Poulsen, J.W., Nielsen, M.L., Bekker-Jensen, S., Mailand, N., and Choudhary, C. (2012). Systems-wide analysis of ubiquitylation dynamics reveals a key role for PAF15 ubiquitylation in DNA-damage bypass. Nat. Cell Biol. *14*, 1089–1098.

Proietti-De-Santis, L., Drané, P., and Egly, J.M. (2006). Cockayne syndrome B protein regulates the transcriptional program after UV irradiation. EMBO J. *25*, 1915–1923.

Randall, R.E., and Goodbourn, S. (2008). Interferons and viruses: an interplay between induction, signalling, antiviral responses and virus countermeasures. J. Gen. Virol. *89*, 1–47.

Rockx, D.A., Mason, R., van Hoffen, A., Barton, M.C., Citterio, E., Bregman, D.B., van Zeeland, A.A., Vrieling, H., and Mullenders, L.H. (2000). UV-induced inhibition of transcription involves repression of transcription initiation and phosphorylation of RNA polymerase II. Proc. Natl. Acad. Sci. USA *97*, 10503–10508.

Schenk, P.W., Boersma, A.W., Brandsma, J.A., den Dulk, H., Burger, H., Stoter, G., Brouwer, J., and Nooter, K. (2001). SKY1 is involved in cisplatin-induced cell kill in Saccharomyces cerevisiae, and inactivation of its human homologue, SRPK1, induces cisplatin resistance in a human ovarian carcinoma cell line. Cancer Res. *61*, 6982–6986.

Schwanhäusser, B., Busse, D., Li, N., Dittmar, G., Schuchhardt, J., Wolf, J., Chen, W., and Selbach, M. (2011). Global quantification of mammalian gene expression control. Nature *473*, 337–342.

Shen, Y.J., Le Bert, N., Chitre, A.A., Koo, C.X., Nga, X.H., Ho, S.S., Khatoo, M., Tan, N.Y., Ishii, K.J., and Gasser, S. (2015). Genome-derived cytosolic DNA mediates type I interferon-dependent rejection of B cell lymphoma cells. Cell Rep. *11*, 460–473.

Sollier, J., and Cimprich, K.A. (2015). Breaking bad: R-loops and genome integrity. Trends Cell Biol. *25*, 514–522.

Subramanian, A., Tamayo, P., Mootha, V.K., Mukherjee, S., Ebert, B.L., Gillette, M.A., Paulovich, A., Pomeroy, S.L., Golub, T.R., Lander, E.S., and Mesirov, J.P. (2005). Gene set enrichment analysis: a knowledge-based approach for interpreting genome-wide expression profiles. Proc. Natl. Acad. Sci. USA *102*, 15545–15550.

Vermeulen, W., and Fousteri, M. (2013). Mammalian transcription-coupled excision repair. Cold Spring Harb. Perspect. Biol. *5*, a012625.

Williams, E.S., Stap, J., Essers, J., Ponnaiya, B., Luijsterburg, M.S., Krawczyk, P.M., Ullrich, R.L., Aten, J.A., and Bailey, S.M. (2007). DNA double-strand breaks are not sufficient to initiate recruitment of TRF2. Nat. Genet. *39*, 696–698, author reply 698–699.

Wilson, M.D., Harreman, M., and Svejstrup, J.Q. (2013). Ubiquitylation and degradation of elongating RNA polymerase II: the last resort. Biochim. Biophys. Acta *1829*, 151–157.

Wolff, S., Weissman, J.S., and Dillin, A. (2014). Differential scales of protein quality control. Cell *157*, 52–64.

Xu, G., Paige, J.S., and Jaffrey, S.R. (2010). Global analysis of lysine ubiquitination by ubiquitin remnant immunoaffinity profiling. Nat. Biotechnol. *28*, 868–873.

Zaidi, M.R., Davis, S., Noonan, F.P., Graff-Cherry, C., Hawley, T.S., Walker, R.L., Feigenbaum, L., Fuchs, E., Lyakh, L., Young, H.A., et al. (2011). Interferon-γ links ultraviolet radiation to melanomagenesis in mice. Nature *469*, 548–553.

Zeman, M.K., and Cimprich, K.A. (2014). Causes and consequences of replication stress. Nat. Cell Biol. *16*, 2–9.

Zhang, F., Ma, T., and Yu, X. (2013). A core hSSB1-INTS complex participates in the DNA damage response. J. Cell Sci. *126*, 4850–4855.

Zhou, Q., Chen, D., Pierstorff, E., and Luo, K. (1998). Transcription elongation factor P-TEFb mediates Tat activation of HIV-1 transcription at multiple stages. EMBO J. *17*, 3681–3691.

Cell Reports, Volume *15*

Supplemental Information

Multiomic Analysis of the UV-Induced DNA Damage Response

Stefan Boeing, Laura Williamson, Vesela Encheva, Ilaria Gori, Rebecca E. Saunders, Rachael Instrell, Ozan Aygün, Marta Rodriguez-Martinez, Juston C. Weems, Gavin P. Kelly, Joan W. Conaway, Ronald C. Conaway, Aengus Stewart, Michael Howell, Ambrosius P. Snijders, and Jesper Q. Svejstrup

<u>Supplemental information:</u>

• 5 Supplementary Figures (Figure S1-S5)

Figure S1, related to Figure 6. Screen performance and pathway enrichment.

Figure S2, related to Figure 6. Enrichment of various pathways in the data sets.

Figure S3, related to Figure 6. Enrichment of ribosome proteins is primarily caused by high scores in the ubiquitylation screens.

Figure S4, related to Figure 6 and 7. ASCC3 protein interacts with RNAPII and CSB.

Figure S5, related to Figure 7. Effect of CSB depletion on global transcription recovery after UV-irradiation.

• Supplementary Figure Legends

• Supplementary Experimental Procedures

• 12 Supplementary Tables (Table S1-S12)

Table S1. CSB interactomes
Table S2. C7ORF26 interactors
Table S3. RNAPII interactomes
Tables S1, S2, and S3 are related to Figure 2.

Table S4. Chromatin proteins
Table S5. Ubiquitylated proteins
Table S6. Phospho-proteome
Tables S4, S5, and S6 are related to Figure 3.

Table S7. RNAi Screen results
Table S7 is related to Figure 5.

Table S8. Ranked phospho-integration
Table S9. Scores by different approaches
Table S10. TC-NER ontology group
Table S11. Pathway enrichment
Table S12. Corum complexes enrichment
Tables S8-S12 are related to Figure 6.

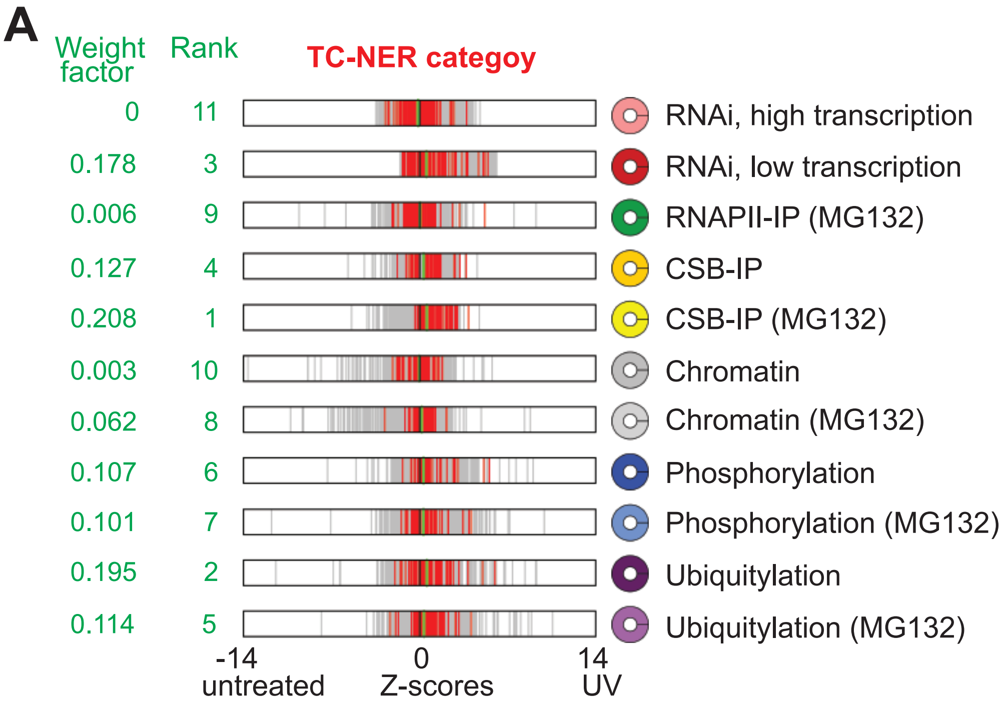
A
Weight factor
Rank
TC-NER categoy
0
11
RNAi, high transcription
0.178
3
RNAi, low transcription
0.006
9
RNAPII-IP (MG132)
0.127
4
CSB-IP
0.208
1
CSB-IP (MG132)
0.003
10
Chromatin
0.062
8
Chromatin (MG132)
0.107
6
Phosphorylation
0.101
7
Phosphorylation (MG132)
0.195
2
Ubiquitylation
0.114
5
Ubiquitylation (MG132)
-14
untreated
0
Z-scores
14
UV

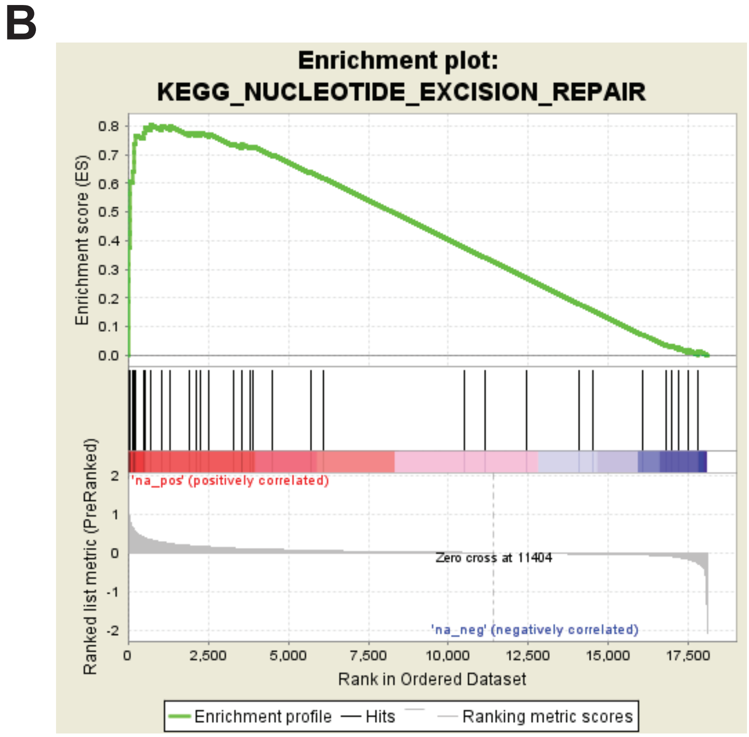
B
Enrichment plot:
KEGG_NUCLEOTIDE_EXCISION_REPAIR
Enrichment score (ES)
Ranked list metric (PreRanked)
'na_pos' (positively correlated)
Zero cross at 11404
'na_neg' (negatively correlated)
Rank in Ordered Dataset
Enrichment profile
Hits
Ranking metric scores

Figure S1

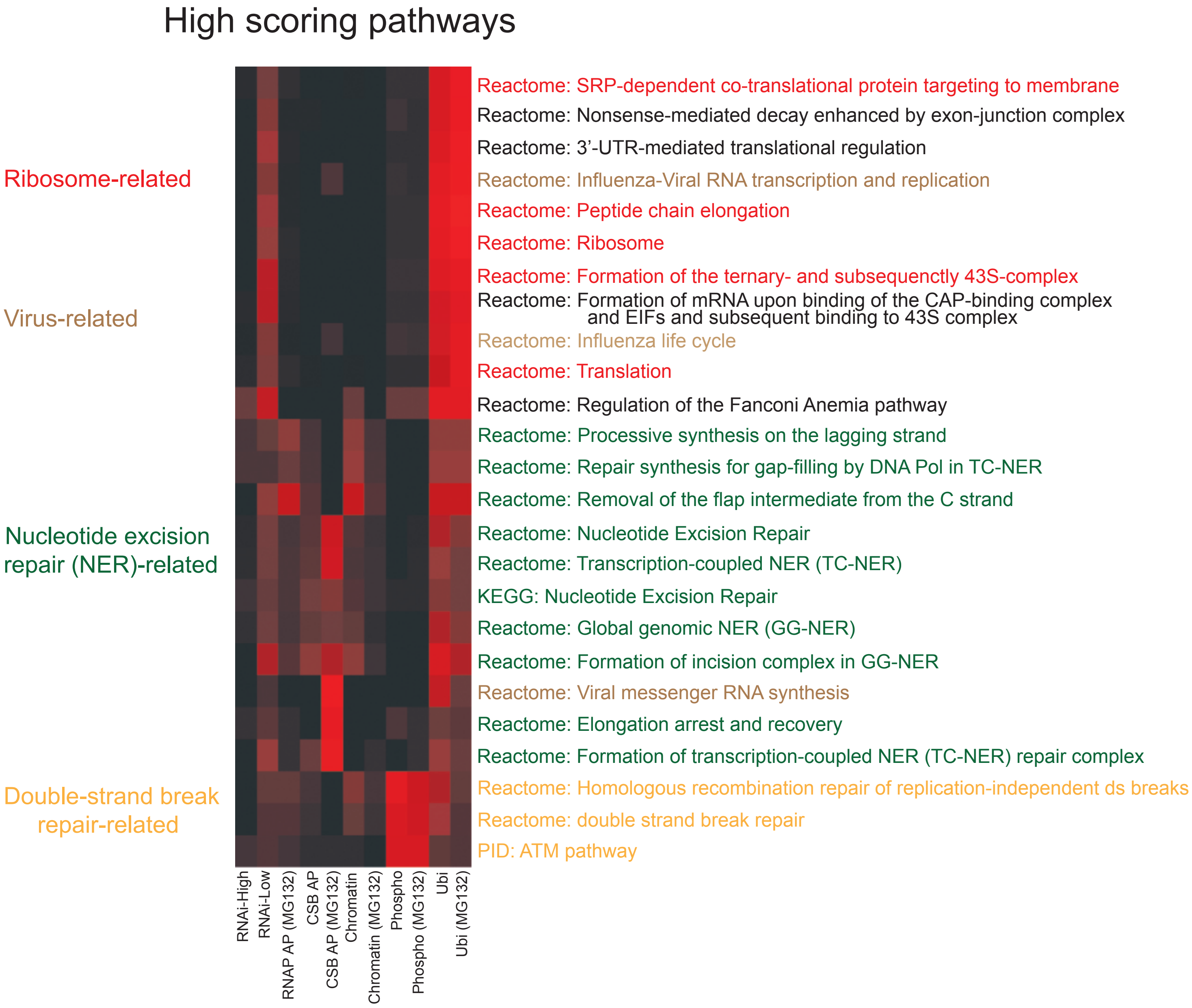
High scoring pathways
Ribosome-related
Virus-related
Nucleotide excision repair (NER)-related
Double-strand break repair-related
Reactome: SRP-dependent co-translational protein targeting to membrane
Reactome: Nonsense-mediated decay enhanced by exon-junction complex
Reactome: 3'-UTR-mediated translational regulation
Reactome: Influenza-Viral RNA transcription and replication
Reactome: Peptide chain elongation
Reactome: Ribosome
Reactome: Formation of the ternary- and subsequenctly 43S-complex
Reactome: Formation of mRNA upon binding of the CAP-binding complex and EIFs and subsequent binding to 43S complex
Reactome: Influenza life cycle
Reactome: Translation
Reactome: Regulation of the Fanconi Anemia pathway
Reactome: Processive synthesis on the lagging strand
Reactome: Repair synthesis for gap-filling by DNA Pol in TC-NER
Reactome: Removal of the flap intermediate from the C strand
Reactome: Nucleotide Excision Repair
Reactome: Transcription-coupled NER (TC-NER)
KEGG: Nucleotide Excision Repair
Reactome: Global genomic NER (GG-NER)
Reactome: Formation of incision complex in GG-NER
Reactome: Viral messenger RNA synthesis
Reactome: Elongation arrest and recovery
Reactome: Formation of transcription-coupled NER (TC-NER) repair complex
Reactome: Homologous recombination repair of replication-independent ds breaks
Reactome: double strand break repair
PID: ATM pathway
RNAi-High
RNAi-Low
RNAP AP (MG132)
CSB AP
CSB AP (MG132)
Chromatin
Chromatin (MG132)
Phospho
Phospho (MG132)
Ubi
Ubi (MG132)

Figure S2

KEGG category
ribosome

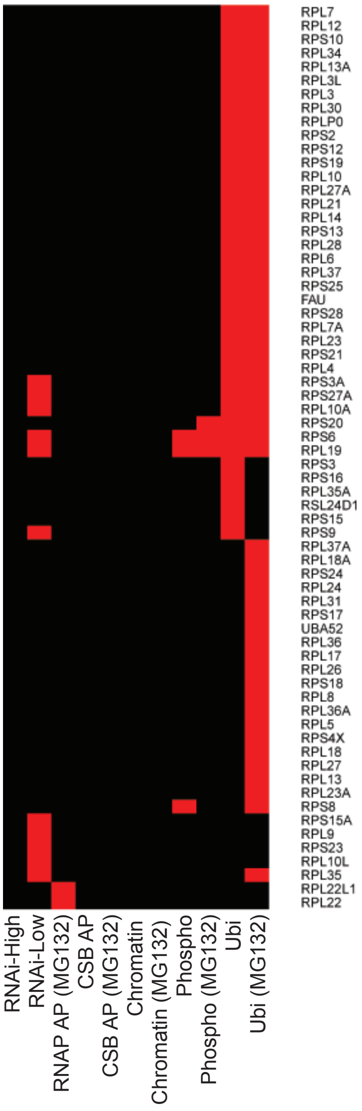

Figure S3

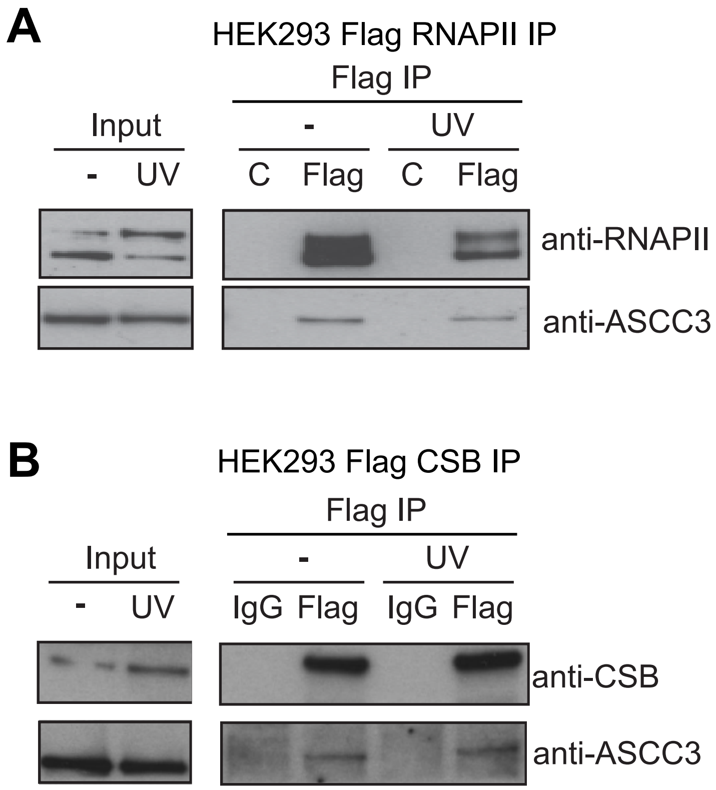
A
HEK293 Flag RNAPII IP
Flag IP
Input
-
UV
- UV
C Flag C Flag
anti-RNAPII
anti-ASCC3
B
HEK293 Flag CSB IP
Flag IP
Input
-
UV
- UV
IgG Flag IgG Flag
anti-CSB
anti-ASCC3

Figure S4

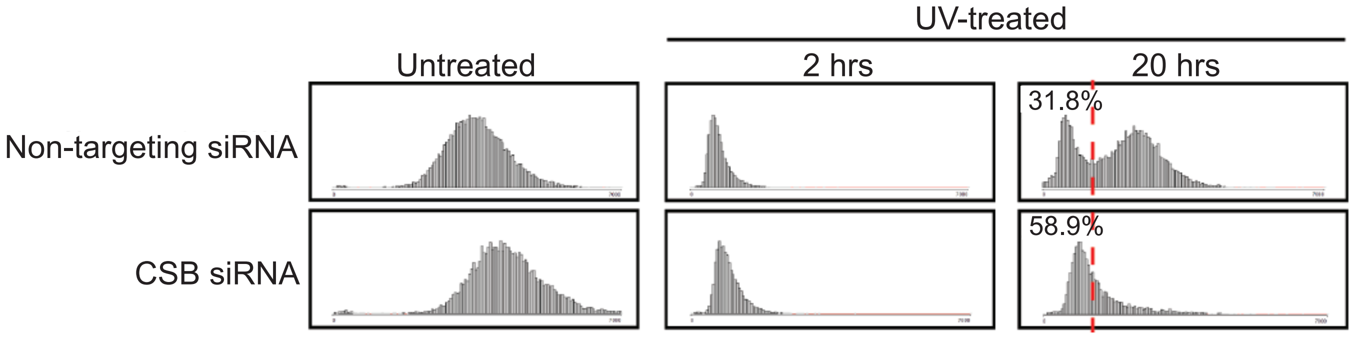
UV-treated
Untreated
2 hrs
20 hrs
Non-targeting siRNA
CSB siRNA
31.8%
58.9%

Figure S5

SUPPLEMENTARY FIGURE LEGENDS

Figure S1, related to Figure 6. **Screen performance and pathway enrichment**. (**A**) The individual screen had different ability to uncover already known TC-NER factors. The median score and the rank of the screens are shown in green. The median score represents the frequency with which proteins from the TC-NER category scores in the indicated screen, on average. The color wheels associated with screens (on the right) are re-used in Figure 6B, C to show relative weight of that screen in the relevant score scheme. (**B**) GSEA category enrichment plot of the KEGG category 'Nucleotide_Excision_Repair' over z score sums.

Figure S2, related to Figure 6. **Enrichment of various pathways in the data sets.** Gene Set Enrichment Analysis (GSEA) used to illustrate the enrichment of pathways related to the ribosome, viruses, NER, and double-strand break repair. See also Table S11.

Figure S3, related to Figure 6. **Enrichment of ribosome proteins is primarily caused by high scores in the ubiquitylation screens.** In contrast to TC-NER and NER proteins, the significant enrichment of ribosome-related proteins is caused primarily by extremely high scores in the ubiquitylation screens, although some also score in the RNAi screen.

Figure S4, related to Figure 6 and 7. **ASCC3 protein interacts with RNAPII and CSB.** Immunoblot showing Flag immunoprecipitation of Flag-tagged RPB3 (**A**) and

Flag-tagged CSB (**B**) from HEK293 cells that were untreated or treated with UV-irradiation (30 J/m^2) followed by a 3 hour incubation. ASCC3 interacted with Flag-RPB3 and CSB equally in the absence and presence of UV-irradiation.

Figure S5, related to Figure 7. **Effect of CSB depletion on global transcription-recovery after UV-irradiation.** In the absence of CSB, a higher percentage of cells remain in the non-transcribing state, even 18-20 hours after DNA damage.

Supplementary Experimental Procedures

Cell culture, UV-irradiation

For CSB-interactomes, gly-gly peptide enrichment, and phospho-peptide enrichments, an inducible TREX 293 FLAG-CSB expressing cell line was used. FLAG-CSB expression was induced 48 hours before UV-irradiation by adding doxycycline at a concentration of 500 ng/ml to the cell culture medium. If indicated, the cell culture medium was supplemented one hour prior to the UV-treatment with the proteasome inhibitor MG132 (Calbiochem) at a concentration of 5 µM.

For RNAPII interactomes and chromatin proteomes, FLAG-RPB3 expressing HEK293 cells (Aygun et al., 2008) were used. Cells were cultured in either R0K0 (light) or R8K10 heavy SILAC medium for at least seven generations. Cells were UV-irradiated with a UV crosslinker (Stratalinker) and allowed to recover at 37°C for three hours prior to extract preparation.

For the ubiquitylome (gly-gly peptide enrichment) and phosphoproteome studies, light and heavy cells were mixed in a 1:1 ratio directly after UV-treatment.

Extract preparation for chromatin extracts and immuno-affinity purifications

The approach taken to purify RNAPII- and CSB-interactors has been described previously (Aygun et al., 2008), with modifications outlined below. Briefly, FLAG-tagged protein was affinity-purified (AP) from Benzonase-treated chromatin, under physiological salt conditions, using anti-FLAG

antibody as previously described (Aygun et al., 2008). Specifically bound proteins were released by competition with FLAG peptide, which dramatically increased detection sensitivity and thus allowed identification of interactors that could not be detected by mass spectrometry after simple SDS-eluted FLAG immunoprecipitation (IP). As a control, we carried out IgG mock-IP experiments. Protein detection intensity was converted into iBAQ values, reflecting absolute protein abundance (Schwanhausser et al., 2011). A protein was deemed to be a specific interactor of CSB (or RNAPII below) if it was detected with at least 10-fold lower abundance in the IgG-mock AP (or not at all, as in the majority of cases). SILAC ratios were log2 transformed and then converted into z-scores in order to allow a standardized comparison to other datasets in the study.

In preparing for purification, cells were harvested in PBS and resuspended in hypotonic buffer (10 mM HEPES-KOH pH 7.5, 10 mM KCl, 1.5 mM $MgCl_2$, protease inhibitors (Roche complete), phosphatase inibitors (Roche PhosStop) and 20 mM N-Ethylmaleimide (NEM). Cells were lysed by dounce homogenization and nuclei pelleted by centrifugation at 3000x g. The cytoplasmic lysate was removed and the nuclei were resuspended in nucleoplasmic extraction buffer (20 mM HEPES pH7.9, 1.5 mM MgCl2, 10% glycerol, 150 mM potassium acetate, 0.05% NP-40, protease and phosphatase inhibitors). After a 20 min incubation on ice, chromatin was pelleted by centrifugation at 20,000x g. The nucleoplasmic lysate was removed and the pellet resuspended in chromatin digestion buffer (20 mM HEPES-KOH 7.9, 1.5 mM $MgCl_2$, 10% glycerol, protease and phosphatase inhibitors as above). The pan-nuclease Benzonase (EMD Millipore) was

added to a concentration of 75 units/ml and nucleic acids digested at 37°C for 15 min. The salt concentration was then increased to 150 mM NaCl by adding a 5 M NaCl solution, dropwise. The NP-40 concentration was similarly brought to 0.05%, and the solution was incubated on ice for 20 min. Debris was then pelleted by centrifugation at 20,000x g and the supernatant collected as chromatin fraction.

All immunoprecipitations in this study were performed using the chromatin fraction. Mouse-IgG coupled beads were used alongside FLAG-M2 beads (Sigma-Aldrich) in each experiment for specificity-control. Both kinds of beads were incubated for 3 hrs at 4°C with chromatin extracts from either FLAG-CSB or FLAG-RPB3 expressing cell lines. AP beads were then washed three times with 50 column-volumes AP-wash buffer (20 mM HEPES pH 7.9, 1.5 mM $MgCl_2$, 10% glycerol, 150 mM NaCl, 0.05% NP-40, protease and phosphatase inhibitors as above), and subsequently eluted with 3xFLAG peptide at 1 mg/ml.

Western blotting

Antibodies for SCAF4 and SCAF8 were purchased from Bethyl, the INTS4 antibody from abcam and the UVSSA antibody from Novus Biologicals. RNAPII CTD serine-2, -5, -7, CTD threonine-4, and CTD tyrosine-1 antibodies were kind gifts of Professor Dirk Eick, University of Munich.

Quantitative diGly Proteomics

Cells were lysed in 9 M urea, 20mM HEPES pH 8.0, supplemented with 100 units/ml of benzonase and sonicated to reduce viscosity (3mm probe, 50 %

amplitude, 3 x 15 sec bursts, on ice). Between 30 - 40 mg of protein per sample were used as estimated by Bradford protein assay. Lysates were reduced with 10 mM dithiothreitol (DTT) (Sigma) for 30 min at room temperature, followed by alkylation with 20 mM chloroacetamide (Sigma) for 30 min at room temperature in the dark. Lysates were digested initially with LysC (Promega) for 2 hours at 37^{0}C. The lysates were then diluted with 100 mM amonium bicarbonate, 5 % acetonitrile to a final urea concentration of less than 2 M. The samples were digested 1:100 enzyme to protein ratio (w/w) with trypsin (Promega) overnight at 37^{0}C. The next day, two additional aliquots of trypsin were added and incubated at 37 ^{0}C four hours each. After the digestion the samples were acidified with TFA (Thermo Fisher Scientific) to final concentration of 1 % (v/v). All insoluble material was removed by centrifugation and the supernatant was desalted with Sep-Pak C_{18} cartridges (Waters) and lyophilized for 2 days.

Peptides containing the diGly remnant were enriched using K-ϵ-GG affinity resin (Cell Signaling Technology) according to the manufacturer's instructions. Briefly, digests were reconstituted in 1.4 ml of immunoaffinity purification (IAP) buffer as supplied by the manufacturer. One aliquot (~40-µl packed bead volume) was washed four times with PBS and mixed with the peptide sample. Incubation of sample and beads was performed with gentle rotation at 4 °C for 2 hours followed by a 30 sec 2000 × *g* spin to pellet the beads. The antibody beads were washed twice with ice-cold IAP buffer followed by three washes with ice-cold water. DiGly peptides were eluted from the beads with the addition of 50 µl of 0.15 % TFA and allowed to stand at room temperature for 5 min. After a 30 sec 2000 × *g* spin, the supernatant was carefully removed

and retained for further analysis. A second 55-μl aliquot of 0.15 % TFA was added to the beads followed by a 30 sec 2000 × *g* spin, and the supernatant was added to the first elution. The eluted peptides were lyophilised for 2 days and used for SCX fractionation.

SCX (strong cationic exchange) fractionation of diGly peptides

For SCX fractionation, peptides eluted from the K-ϵ-GG affinity resin were dissolved in 35 μl of 10 mM Ammonium formate pH 2.9, 25 % acetonitrile. The samples were sonicated and insoluble material was removed by centrifugation. Peptide separation and fraction collection was performed using the micro pump on a RSLCnano U3000 (Thermo Fisher Scientific) at a flow rate of 50 μl/min. The peptides were loaded on 15-cm Polysulfoethyl-Asp SCX column (1 mm inner diameter, 5 μm particle size, PolyLC). Solvent A was 10 mM Ammonium formate pH 2.9, 25 % acetonitrile, and solvent B was 500 mM Ammonium formate pH 6.8, 25 % acetonitrile. The samples were run on a linear gradient of 0-80 % B in 45 min, total run time was 75 min including column conditioning. A total of 30 fractions were collected every minute between 15-45 min after injection (1 fraction= 50 μl). The collected fractions were vacuum dried and used for LC-MS/MS analysis.

SCX fractionation of phosphopeptides

Peptides were resuspended in 90 μl of 10 mM Ammonium formate pH 2.9, 25 % acetonitrile, sonicated and all insoluble material was removed by centrifugation. Separation was performed using Agilent 1200 (Agilent) HPLC at a flow rate of 1 ml/min. The peptides were loaded on 20-cm Polysulfoethyl-

Asp SCX column (4.6 mm inner diameter, 5 µm particle size, PolyLC). Solvent A was 10 mM ammonium formate pH 2.9, 25 % acetonitrile, and the solvent B was 500 mM ammonium formate pH 6.8, 25 % acetonitrile. The samples were run on a linear gradient 0-80 % B in 10 min. The total run time including column conditioning was 30 min. A total of six fractions were collected every two min between 1-13 min after injection (1 fraction = 2 ml). The collected fractions were vacuum dried and used for phosphopeptide enrichment.

TiO2 enrichment of phosphopeptides

Dried fractions were solubilised in 1 ml of loading buffer (80 % acetonitrile, 5 % TFA, 1M glycolic acid), sonicated and mixed with 5 mg of TiO_2 beads (Titansphere, 5 µm GL Sciences Japan). Samples were incubated for 10 min with agitation followed by a 1 min 2000 × *g* spin to pellet the beads. The supernatant was removed and the beads were washed with 150 µl of loading buffer. This was followed by two wash steps, first with 150 µl 80 % acetonitrile, 1 % TFA and second with identical volume of 10 % acetonitrile, 0.2 % TFA. After each wash beads were pelleted by centrifugation (1 min at 2000 × *g*) and the supernatant discarded. The remaining solution and beads were dried in a vacuum centrifuge for 30 min followed by two elution steps at high pH. For the first elution step the beads were mixed with 100 µl of 1 % ammonium hydroxide (v/v), incubated for 10 min with agitation and pelleted at 2000 × *g* for 1 min. For the second elution step the beads were mixed with 100 µl of 5 % ammonium hydroxide (v/v), incubated for 10 min with agitation and pelleted at 2000 × *g* for 1 min. The two elutions were combined, vacuum dried and desalted using C18 Stage Tips (Rappsilber *et al*, 2007, Nat. Prot).

Briefly, each Stage Tip was packed with one C18 Empore™ high performance extraction disk and conditioned with 100 µl of 100 % methanol, followed by 200 µl of 1 % TFA. The sample was loaded on the conditioned Stage Tip, washed six times with 200 µl of 1 % TFA and elute with 50 µl of 80 % acetonitrile, 5 % TFA. The desalted peptides were vacuum dried in preparation for LC-MS/MS analysis.

In-gel digestion

Polyacrylamide gel slices were prepared for mass spectrometric analysis using the Janus liquid handling system (Perkin-Elmer). Briefly, the excised protein gel piece was placed in a well of a 96-well microtiter plate and destained with 50 % acetonitrile, 50 mM ammonium bicarbonate, reduced with 10 mM DTT, and alkylated with 55 mM iodoacetamide. After alkylation, the proteins were digested with 6 ng/µl trypsin overnight at 37°C. The resulting peptides were extracted in 2 % formic acid, 1 % acetonitrile.

LC-MS/MS

For MS analysis, peptides were resuspended in 0.1 % TFA and loaded on 50-cm Easy Spray PepMap column (75 µm inner diameter, 2 µm particle size, Thermo Fisher Scientific) equipped with an integrated electrospray emitter. Reverse phase chromatography was performed using the RSLC nano U3000 (Thermo Fisher Scientific) with a binary buffer system at a flow rate of 250 nl/min. Solvent A was 0.1 % formic acid, 5 % DMSO, and solvent B was 80 % acetonitrile, 0.1 % formic acid, 5 % DMSO. The diGly enriched samples and the in-gel digested samples (RNA polymerase IP, CSB IP and chromatin

proteome) were run on a linear gradient of solvent B (2- 40 %) in 90 min, total run time of 120 min including column conditioning. The phopsho-enriched samples were run on a linear gradient of 2-35 % B in 150 min, total run time of 186 min. The nanoLC was coupled to a Q Exactive mass spectrometer using an EasySpray nano source (Thermo Fisher Scientific).

The Q Exactive was operated in data-dependent mode acquiring HCD MS/MS scans (R=17,500) after an MS1 scan (R=70, 000) on the 10 most abundant ions using MS1 target of 1×10^6 ions, and MS2 target of 5×10^4 ions. The maximum ion injection time utilized for MS2 scans was 120 ms, the HCD normalized collision energy was set at 28, the dynamic exclusion was set at 10 s, and the peptide match and isotope exclusion functions were enabled.

The phospho-enriched samples were also analysed using a LTQ Orbitrap Velos (Thermo Fisher Scientific) where the HPLC conditions were as described above. CID and Multi Stage Activation (MSA) fragmentation were used in separate runs to increase the total number of identified phosphopeptides. The LTQ Orbitrap Velos was operated in data-dependent mode acquiring 10 CID or MSA MS2 scans (R=17,500) after an MS1 scan (R=60, 000). MS1 target was set at 1×10^6 ions, and MS2 target at 3×10^4 ions. The CID normalized collision energy was set at 35 with 10 ms activation time and a maximum ion injection time for MS2 scans at 50 ms. The dynamic exclusion was set at 20 s and singly charged peptides and peptides with unassigned charge states were excluded from fragmentation.

Proteomics data processing and analysis

Raw data files were analysed with MaxQuant software (version 1.3.0.5) as described previously (Cox et al., 2009). Parent ion and tandem mass spectra were searched against UniprotKB *Homo sapiens* database (August 2012). A list of 247 common laboratory contaminants provided by MaxQuant was also added to the database. For the search the enzyme specificity was set to trypsin with maximum of three missed cleavages for the diGly dataset and two missed cleavages for the rest of the data. The precursor mass tolerance was set to 20 ppm for the first search (used for mass re-calibration) and to 6 ppm for the main search. Carbamidomethylation of cysteines was specified as fixed modification, oxidized methionines and N-terminal protein acetylation were searched as variable modifications. Di-glycine-lysine or phosphorylated serine, threonine and tyrosine were added to the list of variable modifications when samples enriched for ubiquitinated or phosphorylated peptides were searched. The datasets were filtered on posterior error probability to achieve 1 % false discovery rate on protein, peptide and site level.

Proteomic raw data were processed using the MAXQUANT software package (Cox et al., 2009). Log2-transformed Heavy to Light SILAC ratios (Maxquant output) were converted into z-scores as follows:

SILAC Z-score = [log2(SILAC Ratio H/L) – mean of all log2(SILAC Ratio H/L)] / standard deviation (all log2(SILAC Ratio H/L)). Ensembl gene identifiers were added using the PERSEUS software package (Cox and Mann, 2012). Based on the Ensembl gene identifiers, official human genome nomenclature (HGNC) gene names were added. Phosphorylation and Ubiquitylation data was processed in a similar way. Official HGNC gene symbols were assigned to siRNAs data using Ensembl gene identifiers.

Individual proteomic and RNAi datasets were intersected using ensemble gene identifiers. Aggregated z-scores were calculated by adding up the z-scores of the individual experiments. In case a protein had more than one phosphorylation or more than one ubiquitylation site in any one experiment, only the highest scoring phosphorylation and/or ubiquitylation site was considered for that particular experiment when calculating aggregated z-scores.

RNAi Screen

Dharmacon Human siGENOME library was aliquoted into 384-well plate format. Each well contained a pool of 4 siRNAs targeting a single gene. siRNAs targeting CSB and the RPB1 subunit of RNAPII were included as positive controls and RISC-free and non-targeting pool 2 as negative controls. siRNA pools diluted in HBSS (Invitrogen) (5µL/well, final concentration 37.5 nM) and the transfection reagent INTERFERin (Polyplus-transfection) diluted in Opti-MEM (Invitrogen) (5 µL/well, final concentration of 0.05 µl/well) were deposited in clear-bottomed 384-well plates (Greiner Bio One). After 15 min incubation at room temperature, MRC5VA cells were diluted in DMEM supplemented with 10% fetal bovine serum and 5% penicillin/streptomycin and deposited on top of the transfection mix (900 cells/well) to give a final volume of 50 µL per well. Plates were incubated at 37°C and 5% CO_2 for 48 hour. Media was removed from the plates and the plates were exposed to UVC light (TUV 16W G16 T5, Philips) using a purpose built UV irradiation machine that enabled an even exposure of UV light across the plate (stationary bulb coupled to a conveyor belt that moved the plate

under the bulb in a constant manner). Media was then added back onto the plates and they were incubated for a subsequent 18 hours. Media was then removed from the plates and replaced with fresh media containing 0.75 mM 5'-ethynyl uridine (Jena Bioscience). Plates were incubated with 5'-ethynyl uridine containing media for 2 hour to allow for uptake into the cells and incorporation in the nascent RNA. Media was then removed and the cells were fixed with 3.7% formaldehyde (Sigma) for 60 min at room temperature followed by one wash with PBS and permeabilization using 0.5% TX-100 for 30 min. Plates were then washed once with PBS followed by incubation with staining solution (100 mM Tris pH 8.5 (Sigma), 4 mM $CuSO_4$ (Sigma), 10 µM AlexaFlour azide 488 nM (Invitrogen), 100 mM ascorbic acid (Sigma)) for 60 min at room temperature. Plates were washed 3 times in Tris pH7.5 and stained with DAPI (4′,6-diamidino-2-phenylindole dihydrochloride, Invitrogen) at a final concentration of 1 µg/ml. Plates were washed once with PBS. The screen was performed in triplicate. Automated image acquisition of 6 fields per well was performed on a Cellomics Array Scan VTI HCS reader (Thermo Scientific) using a 10× objective. Image analysis was performed using HCS Studio (Thermo Scientific). Cell nuclei were masked using the DAPI staining. The average intensity of AlexaFluor-conjugated 5' ethynyl uridine-labeled RNA was measured for each nucleus. The high transcription score was calculated by taking the average fluorescence intensity per nuclei per well for each replicate and then normalizing to the plate median. The normalised values were then converted to a z-score and the median of the 3 replicate z scores was used as the final score. We set an arbitrary cutoff of z= 2.0 to classify siRNA hits as high transcribers. The low transcription score was

calculated by determining the percentage of cells per well below a defined threshold and normalizing it to the plate median. The normalized values were then converted to a z score and the median of the 3 replicate z scores was used as the final score. We set an arbitrary cutoff of z=2.5 to classify siRNA hits as low transcribers.

Statistical evaluation

Category enrichment p-values for pathways and protein complexes were calculated using the Kolmogorov-Smirnov test.

Data sources

Protein complex data was retrieved from CORUM (Ruepp et al., 2010), protein-protein interaction data from biogrid (Stark et al., 2006), protein domain data from INTERPRO (http://www.ebi.ac.uk/interpro), pathways were taken from Reactome (http://www.reactome.org), the pathway interaction database (http://pid.nci.nih.gov) KEGG (http://www.genome.jp/kegg/pathway.html) and mySigDB (Subramanian et al., 2005).

Aggregate scores

Point scores: Each gene earned one point for each screen in which it had scored above the z-score cut-off. For the RNAi low screen that z- score cut off was set arbitrarily to 2.5; for the RNAi high screen to 2 and for all proteomic screens to 1.5. If a gene featured more than one phosphorylation and/or ubiquitylation site, only the highest scoring site was used to calculate the z-

score sum for that gene in the respective screen. The median score among proteins of the TC-NER reference category in the point scoring scheme was 1 using this approach (compared to 0 for a list of all curated proteins), indicating an enrichment of the benchmark category in the overall data (Category enrichment p-value=$1.69*10^{-14}$).

Z-score sums: Z-score sums were calculated by adding up the z-scores of individual screens for each gene. In case a gene had not featured in a particular screen, 0 was added to the total z-score sum. If a gene featured more than one phosphorylation and/or ubiquitylation site, only the highest scoring site was used to calculate the z-score sum for that gene in the respective screen. The median of the TC-NER benchmark category in this metric was at 0.17, indicating an enrichment of this category (category enrichment p-value < $2.2*10^{-16}$).

Weighted z-score sums: For the weighted z-score sums the z-scores of the individual screens were multiplied with a weight factor proportional to the scores of the TC-NER training category in that screen. The weight factors were as follows: RNAi high screen: 0; RNAi low screen: 0.178; RNAPII-IP(MG132): 0.006; CSB-IP: 0.127; CSB-IP(MG132): 0.208; chromatin MS: 0.003; chromatin MS(MG132): 0.062; phosphorylation: 0.107; phosphorylation (MG132): 0.101; ubiquitylation: 0.195; ubiquitylation(MG132): 0.114. Weighted z-scores were summed up for each gene in the same way as for the z-score sums above. In this metric the median of the TC-NER benchmark

category was 0.41, the median score for all other genes was 0.02 (category enrichment p-value < $2.2*10^{-16}$)

Protein Kinase networks

In order to infer possible kinase-substrate relationships, we took advantage of curated protein-protein interaction data in the Biogrid database (www.thebiogrid.org). Based on this, we created a protein-protein interaction network and analyzed if proteins, that are directly connected to a protein kinase exhibit an increased phosphorylation status (defined as a z-score of 1.5 or bigger in at least one of the phosphor proteomic experiments) upon UV-irradiation.

Laser micro-irradiation microscopy

Cells were plated in gridded MatTek dishes (35mm, No. 2 14mm diameter glass) at a density of 2 x 10^6 per dish in media containing DMEM + 10% FCS + 100 µg/ml Hygromycin B + 15 µg/ml blasticidin supplemented with 5 µg/mL doxycycline hyclate (Sigma-Aldrich D9891) for 48 hours. 30 min prior to imaging, culture medium was replaced with phenol-red free medium containing the same additives plus 1 µg/ml Hoechst 33258 to label nuclei and sensitize cells to UV irradiation.

UV-microirradiation was performed by subjecting cell nuclei to laser micro-irradiation in a 200 x 3 pixel (34 x 0.51 µm) stripe or a diffraction limited spot. Micro-irradiation was performed with 100% 405 nm laser power and cells were exposed to 500-700 µW for approximately 3s (40 iterations) and

1.5s (250 iterations) for the stripe and spot, respectively. Normal cell and nuclear morphology were preserved over the time scale of the experiment.

Micro-irradiation and imaging was performed on a Perkin Elmer UltraVIEW VoX spinning disk microscope, which included a Yokagawa CSU-X11 spinning disk, an ORCA-R2 camera (Hamamatsu), and a Perkin Elmer PhotoKinesis accessory. The microscope base was a Carl Zeiss Axiovert 200M equipped with a 40x 1.3 NA Plan-Apochromat objective and a 37° C, 5% CO_2 incubator (Solent Scientific). STK19-GFP was excited with the 488 nm laser and imaged with a 500-550 emission filter. Laser power and exposure time were adjusted before-hand to maximize image quality and minimize photobleaching; absence of significant photobleaching was confirmed by observing unperturbed cells in the acquisition field of view.

References:

Aygun, O., Svejstrup, J., and Liu, Y. (2008). A RECQ5-RNA polymerase II association identified by targeted proteomic analysis of human chromatin. Proceedings of the National Academy of Sciences of the United States of America *105*, 8580-8584.

Cox, J., and Mann, M. (2012). 1D and 2D annotation enrichment: a statistical method integrating quantitative proteomics with complementary high-throughput data. BMC Bioinformatics *13 Suppl 16*, S12.

Cox, J., Matic, I., Hilger, M., Nagaraj, N., Selbach, M., Olsen, J.V., and Mann, M. (2009). A practical guide to the MaxQuant computational platform for SILAC-based quantitative proteomics. Nat Protoc *4*, 698-705.

Ruepp, A., Waegele, B., Lechner, M., Brauner, B., Dunger-Kaltenbach, I., Fobo, G., Frishman, G., Montrone, C., and Mewes, H.W. (2010). CORUM: the comprehensive resource of mammalian protein complexes--2009. Nucleic acids research *38*, D497-501.

Schwanhausser, B., Busse, D., Li, N., Dittmar, G., Schuchhardt, J., Wolf, J., Chen, W., and Selbach, M. (2011). Global quantification of mammalian gene expression control. Nature *473*, 337-342.

Stark, C., Breitkreutz, B.J., Reguly, T., Boucher, L., Breitkreutz, A., and Tyers, M. (2006). BioGRID: a general repository for interaction datasets. Nucleic acids research *34*, D535-539.

Subramanian, A., Tamayo, P., Mootha, V.K., Mukherjee, S., Ebert, B.L., Gillette, M.A., Paulovich, A., Pomeroy, S.L., Golub, T.R., Lander, E.S., *et al.* (2005). Gene set enrichment analysis: a knowledge-based approach for interpreting genome-wide expression profiles. Proceedings of the National Academy of Sciences of the United States of America *102*, 15545-15550.
